# Loss of hepatic chaperone-mediated autophagy accelerates proteostasis failure in aging

**DOI:** 10.1111/acel.12310

**Published:** 2015-01-23

**Authors:** Jaime L Schneider, Joan Villarroya, Antonio Diaz-Carretero, Bindi Patel, Aleksandra M Urbanska, Mia M Thi, Francesc Villarroya, Laura Santambrogio, Ana Maria Cuervo

**Affiliations:** 1 Department of Developmental and Molecular Biology, Albert Einstein College of Medicine1300 Morris Park Ave, Bronx, NY, 10461, USA; 2 Institute for Aging Studies, Albert Einstein College of Medicine1300 Morris Park Ave, Bronx, NY, 10461, USA; 3 Hospital de la Santa Creu i Sant PauAv. Sant Antoni Maria Claret 167, Barcelona, 08025, Spain; 4 Department of Pathology, Albert Einstein College of Medicine1300 Morris Park Ave, Bronx, NY, 10461, USA; 5 Department of Neuroscience, Albert Einstein College of Medicine1300 Morris Park Ave, Bronx, NY, 10461, USA; 6 Department of Biochemistry and Molecular Biology, University of Barcelona, and CIBER Fisiopatologia Obesidad y NutriciónAv. Diagonal, 643, Barcelona, 08028, Spain

**Keywords:** autophagy, lysosomal protein degradation, macroautophagy, oxidative stress, proteotoxicity, protein aggregation, ubiquitin-proteasome system

## Abstract

Chaperone-mediated autophagy (CMA), a cellular process that contributes to protein quality control through targeting of a subset of cytosolic proteins to lysosomes for degradation, undergoes a functional decline with age. We have used a mouse model with liver-specific defective CMA to identify changes in proteostasis attributable to reduced CMA activity in this organ with age. We have found that other proteolytic systems compensate for CMA loss in young mice which helps to preserve proteostasis. However, these compensatory responses are not sufficient for protection against proteotoxicity induced by stress (oxidative stress, lipid challenges) or associated with aging. Livers from old mice with CMA blockage exhibit altered protein homeostasis, enhanced susceptibility to oxidative stress and hepatic dysfunction manifested by a diminished ability to metabolize drugs, and a worsening of the metabolic dysregulation identified in young mice. Our study reveals that while the regulatory function of CMA cannot be compensated for in young organisms, its contribution to protein homeostasis can be handled by other proteolytic systems. However, the decline in the compensatory ability identified with age explains the more severe consequences of CMA impairment in older organisms and the contribution of CMA malfunction to the gradual decline in proteostasis and stress resistance observed during aging.

## Introduction

In chaperone-mediated autophagy (CMA) (Cuervo, 2010; Kaushik & Cuervo, 2012), cytosolic proteins bearing a targeting motif biochemically related to the pentapeptide KFERQ (Dice, 1990) are recognized by the chaperone hsc70 that delivers them one-by-one to lysosomes for degradation (Chiang *et al*., 1989). Chaperone-mediated autophagy is distinct from other types of autophagy in that it does not require sequestration of the proteins to be degraded (cargo) into any type of vesicles for lysosomal delivery. Instead, a transmembrane protein, the lysosome-associated membrane protein type 2A (LAMP-2A) (Cuervo & Dice, 1996), serves as both the CMA lysosomal receptor to which substrate proteins bind, and the main component of the complex that facilitates their translocation into the lysosomal lumen (Bandyopadhyay *et al*., 2008). Chaperone-mediated autophagy function declines with age (Cuervo & Dice, 2000) and is also compromised in diseases such as neurodegenerative conditions, metabolic disorders, and cancer (Cuervo, 2010; Kaushik & Cuervo, 2012).

Among the estimated 30% of cytosolic proteins that contain a CMA targeting motif, validated CMA substrates include proteins involved in transcriptional regulation (Aniento *et al*., 1996), protein degradation (Cuervo *et al*., 1995b), neuronal survival and function (Cuervo, 2004), hypoxia (Hubbi *et al*., 2013), oncogenesis (Vakifahmetoglu-Norberg *et al*., 2013), and metabolism (Schneider *et al*., 2014). Selective degradation of these proteins by CMA contributes to the tight regulation of their cellular levels and, consequently, of the cellular processes in which they participate. For example, recent studies from our group have revealed that timely degradation by CMA of liver enzymes involved in glycolysis and lipogenesis is required for metabolic adaptation to nutritional changes (Schneider *et al*., 2014).

In addition to this regulatory role, previous studies have shown that CMA is activated in response to stressors such as nutrient deprivation (Cuervo *et al*., 1995a), oxidative stress (Kiffin *et al*., 2004), acute lipid overload (Rodriguez-Navarro *et al*., 2012), and proteotoxicity (Cuervo, 2004; Koga *et al*., 2011a). Chaperone-mediated autophagy exerts a protective function in these conditions by selectively targeting damaged or misfolded proteins for lysosomal degradation. Consistent with this role in protein quality control, blockage of CMA in cultured cells renders them more susceptible to oxidative stress (Massey *et al*., 2006), whereas genetic manipulation that prevents the age-dependent decrease of CMA in mouse liver markedly reduces the content of oxidized proteins in this organ and improves overall hepatocyte homeostasis and function in old animals (Zhang & Cuervo, 2008). However, the specific function(s) of CMA that contributes to delaying hepatic aging remains unknown.

Interestingly, despite the well-supported contribution of CMA to the maintenance of protein homeostasis, our recent studies using a novel mouse model with defective CMA in liver (conditional deletion of LAMP-2A, L2A) revealed that proteostasis was preserved in this organ in young mice (Schneider *et al*., 2014). Previous studies in cultured cells have shown that different protein degradation systems are able to compensate for one another (Iwata *et al*., 2005; Korolchuk *et al*., 2009). This compensation also applies to CMA as its blockage in fibroblasts leads to increased macroautophagy (Massey *et al*., 2006), whereas macroautophagy compromise results in constitutive CMA activation (Kaushik *et al*., 2008). It is not known whether compensation of other proteolytic systems in response to CMA failure occurs *in vivo* and whether this activation is sustained with age.

In this work, using a mouse model with hepatic deletion of L2A, we have examined how loss of CMA impacts protein quality control *in vivo* and how other proteolytic systems react to the loss of this form of selective autophagy. We have found that macroautophagy and proteasomal pathways can compensate for hepatic CMA blockage in young mice, explaining their preserved basal proteostasis. However, these compensatory responses are diminished upon infliction of stress or during aging. Decompensation has negative consequences for the maintenance of proteostasis in old mice. These findings show, for the first time, that basal hepatic proteostasis can be assumed by other proteolytic systems upon CMA loss *in vivo* but that this compensatory activation is lost with age, highlighting the need for intact CMA activity to sustain protein homeostasis in the aging liver.

## Results

### Proteolytic systems compensate for loss of hepatic CMA in young mice

We have recently shown that *in vivo* blockage of CMA in liver, by conditional deletion of LAMP-2A (L2A) in this organ (Albumin-Cre:L2A^f/f^ mice, hereafter referred to as L2AKO mice), leads to alterations in glucose and lipid metabolism due to loss of the regulation of hepatic metabolic enzymes levels by CMA (Schneider *et al*., 2014). In contrast, we did not observe significant differences in hepatic protein homeostasis (i.e., levels of aggregates and oxidized proteins) between control (Ctr) and L2AKO young mice, suggesting that other cellular mechanisms may compensate for the well-documented role of CMA in protein quality control.

**Figure 1:**
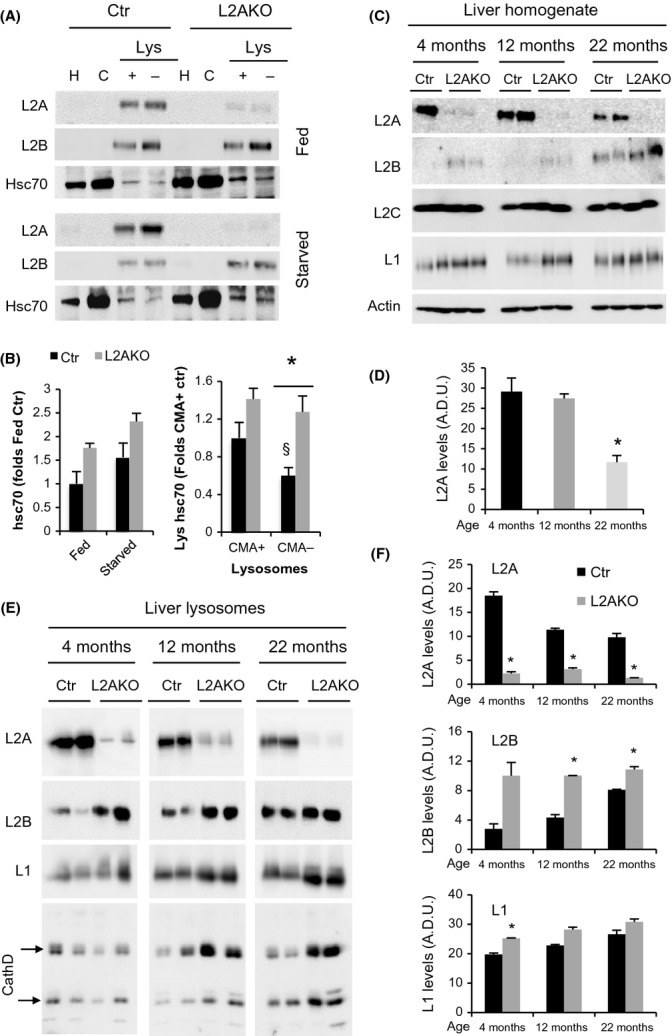
Lysosomal changes in CMA-deficient mice mimic those in old mice livers. (A) Immunoblot (IB) of homogenate (H), cytosol (C), and lysosomes (Lys) with high (+) or low (−) CMA activity isolated from livers of fed or 24 h starved control (Ctr) or Albumin-Cre:L2A^f/f^ (L2AKO) mice. Internal controls for the same preparations are shown in Fig. S1A. (B) Quantifications of cytosolic (left) (*n* = 4) and lysosomal (right) (*n* = 9) hsc70. (C-F) IB of homogenate (C) and CMA-active lysosomes (E) from 24 h starved Ctr and L2AKO mice of the indicated ages. Quantifications for homogenate (*n* = 4) and lysosomes (*n* = 2) are shown in D and F, respectively. All values are expressed as mean ± SEM. Differences with Ctr (*) or with CMA+ lysosomes (§) are significant for *P < 0.05.

Previous studies have identified one such compensatory mechanism that attempts to sustain CMA activity by increasing the fraction of cellular lysosomes dedicated to this pathway. The major cause of CMA functional decline with age is the loss of L2A stability at the lysosomal membrane. However, the decline in L2A levels is initially associated with an increase in the percentage of lysosomes positive for hsc70 (the chaperone that mediates substrate translocation into lysosomes), in an effort to maximize net degradation through CMA, even in the context of lower L2A levels (Cuervo & Dice, 2000; Kiffin *et al*., 2007). To determine whether genetic elimination of L2A causes a similar response in young animals, we isolated two previously characterized populations of lysosomes (with either high (+) or low (−) CMA activity (Cuervo *et al*., 1997)) from livers of control (Ctr) and L2AKO mice. We observed that both total and lysosomal levels of hsc70 were higher in L2AKO mice livers compared to Ctr and that the population of CMA-inactive lysosomes in L2AKO mice displayed a marked increase in lysosomal hsc70 (Figs1A,B and S1A). The residual levels of L2A observed in L2AKO mice originate from liver cells other than hepatocytes (i.e., Kupffer cells, endothelial cells), as demonstrated by immunohistochemistry for L2A in liver sections (Fig. S1B).

To identify further lysosomal changes reactive to the reduction of L2A levels that may mimic changes observed during aging, we analyzed livers from Ctr and L2AKO mice at different ages. As previously described, Ctr animals showed a gradual reduction in total (Fig.1C,D) and lysosomal (Fig.1E,F) L2A levels with age (Cuervo & Dice, 2000), decreasing by more than 50% at 22 month. In contrast, levels of the other two splice variants of the *lamp2* gene were either unchanged (i.e., L2C) or, in the case of L2B, markedly increased in L2AKO mice throughout their lifespan (Fig.1C-F). Interestingly, this compensation by L2B was also noticeable in livers from the oldest Ctr group (Fig.1C-F), supporting that increased L2B levels in young L2AKO mice mimic the changes in L2B that occur with age. As reducing L2A in young mice induces changes in the lysosomal compartment that phenocopies those that occur with age (increase in lys-hsc70 and L2B), L2AKO animals could be a useful model to study possible compensatory mechanisms elicited by loss of CMA during physiological aging independently of other age-dependent changes.

**Figure 2:**
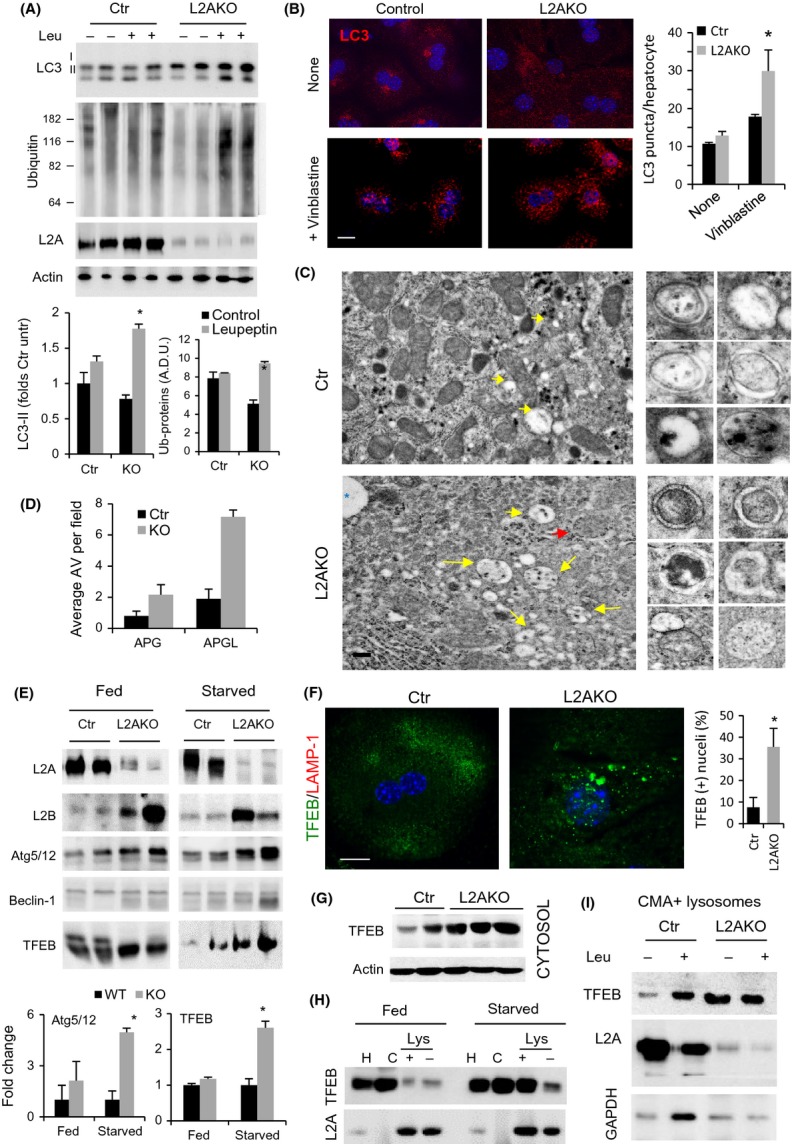
Macroautophagy compensates for CMA failure in young mice livers. (A) IB of liver homogenates from 4-month-old Ctr and L2AKO mice i.p. injected with leupeptin 2 h prior tissue dissection. Bottom: quantification, *n* = 4. (B) Immunofluorescence of LC3 in hepatocytes from Ctr and L2AKO mice treated or not with vinblastine. Right: quantification of average puncta per cell, *n* = 6 (scale bar: 10 μm). (C) Ultrastructure of livers from Ctr and L2AKO mice. Arrows indicate autophagosomes (APG, red) or autophagolysosomes (APGL, yellow). Inserts on the right show examples of both types of vesicles. Additional fields are shown in Fig. S2A. (scale bar: 5 μm) (D) Morphometric quantification of APG and APGL,*n* = 4. (E) IB for a panel of macroautophagy-related proteins of livers homogenates from fed or 24 h starved Ctr and L2AKO mice. Bottom: densitometric quantification, *n* = 4. (F) Immunofluorescence for TFEB (green) in primary hepatocytes isolated from 4-month-old Ctr and L2AKO mice (scale bar: 10 μm). Right: quantification of percentage of cells with nuclear TFEB signal, *n* = 6. Full filed images are shown in Fig. S2C. (G) IB for TFEB in cytosolic fractions from Ctr and L2AKO mice. (H) IB of homogenates (H), cytosol (C), and lysosomes active (+) or inactive (−) for CMA isolated from livers of fed or 24 h starved Ctr and L2AKO mice. (I) IB of isolated lysosomes active for CMA (CMA+) isolated from livers of Ctr and L2AKO mice treated or not with leupeptin 2 h prior tissue dissection. All values are expressed as mean ± SEM. Differences are significant for *P < 0.05.

Using this model, we set out to identify the hepatocyte reaction to CMA failure *in vivo* by analyzing changes in other proteolytic systems in livers of L2AKO mice. Our previous analysis of L2AKO mice demonstrated that even with complete ablation of CMA in the liver, other lysosomal functions and autophagic pathways, such a macroautophagy, were fully preserved (Schneider *et al*., 2014), leaving open the possibility that they could compensate for CMA loss. To compare macroautophagy activity in Ctr and L2AKO livers, we analyzed the degradation of LC3 (LC3 flux), a constituent of autophagic vacuoles that is degraded along with the macroautophagy cargo in lysosomes (Tanida *et al*., 2005). We observed increased degradation rates of LC3-II and higher turnover of the fraction of ubiquitinated proteins degraded in lysosomes (Fig.2A) in L2AKO mice *in vivo* upon blockage of lysosomal degradation by intraperitoneal injection of leupeptin (Fig.2A). Immunofluorescence staining in isolated hepatocytes revealed a comparable steady-state number of LC3 positive puncta in hepatocytes from Ctr and L2AKO mice, but treatment with vinblastine (to prevent autophagosome/lysosome fusion) leads to a significantly higher increase in LC3 puncta in L2AKO cells, in further support of enhanced autophagic flux (Fig.2B). In agreement with enhanced macroautophagy, morphometric analysis of electron micrographs of livers from Ctr and L2AKO mice revealed a significant increase in the number of both autophagosomes but, more so, of autophagolysosomes in CMA-deficient livers (Figs2C,D and S2A). Together, these data confirm that macroautophagy is upregulated in response to loss of CMA in young mice livers.

To further investigate the basis for macroautophagy upregulation in livers of L2AKO mice, we compared levels of proteins that participate in different steps of macroautophagy: transcriptional regulation (TFEB), initiation of autophagosome formation (Beclin-1), cargo recognition (p62 and NBR1), elongation of the limiting autophagosome membrane (Atg5/12), and autophagosomelysosome fusion (LAMP-2B) (Yang & Klionsky, 2010). In agreement with a fully functional macroautophagy-dependent degradation, levels of cargo receptors were slightly reduced in L2AKO mice livers (Fig. S2B). Besides the increase in LAMP-2B levels, Atg5/12 and TFEB also increased significantly in starved L2AKO mice livers (Fig.2E). TFEB has been described as the master regulator of macroautophagy because, upon nuclear translocation, it activates the transcriptional program that controls this autophagic pathway (Settembre *et al*., 2011). Using immunofluorescence (Figs2F and S2C) and cellular subfractionation (Figs2G and S2D-F), we confirmed increased levels of TFEB in both cytosol (3.19 + 0.15 folds) and also nucleus (1.85 + 0.16 folds) in L2AKO mice livers and a higher percentage of cells with detectable nuclear TFEB (Fig. S2C). Interestingly, we identified two conserved CMA targeting motifs in the TFEB sequence (Fig. S2G) and found preferential association of TFEB with lysosomes active for CMA during starvation (Fig.2H) (when CMA is maximally activated), suggestive of the fact that TFEB could undergo degradation via CMA. Analysis of lysosomes isolated from Ctr mice injected with leupeptin confirmed that, upon prolonged starvation, a fraction of cellular TFEB does undergo degradation in CMA-active lysosomes (Fig.2I). Similar studies in L2AKO mice demonstrated that despite higher association of TFEB to lysosomes in these animals, degradation of TFEB in this compartment was completely prevented, supporting that active CMA is required for the observed lysosomal degradation of TFEB (Fig.2I). Overall, our findings indicate that *in vivo* blockage of CMA leads to upregulation of macroautophagy attained, at least in part, through the increase in levels of TFEB due to its reduced turnover by CMA. Selective degradation of TFEB by CMA may underlie the basis of the cross-talk between these two stress-induced autophagic pathways.

**Figure 3:**
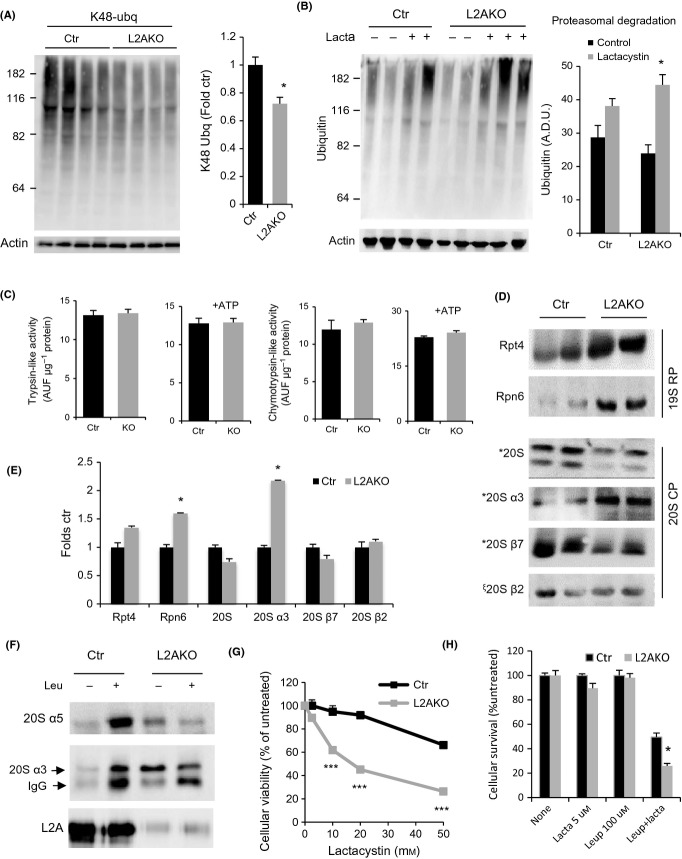
Enhanced ubiquitin-proteasome activity in livers from young CMA-deficient mice. (A) Immunoblot (IB) for K48-linked ubiquitinated proteins of liver homogenates from 4-month-old control (Ctr) and Albumin-Cre:L2A^f/f^ (L2AKO) mice. Right: densitometric quantification, *n* = 10. (B) IB of Ctr and L2AKO mouse livers incubated for 2 h in the presence/absence of lactacystin (lacta). Right: densitometric quantification, *n* = 4. (C) Proteasome activity against fluorogenic substrates specific for trypsin- or chymotrypsin-like activities measured in the presence or absence of ATP in liver homogenates from Ctr and L2AKO mice, *n* = 6. (D) IB for subunits of the proteasome core (20S) or regulatory (19S) particles of 4-month-old Ctr and L2AKO mice livers. (E) Densitometric quantification of blots as in D, *n* = 3. (F) IB of isolated lysosomes active for CMA isolated from livers of 4-month-old Ctr and L2AKO mice treated or not with leupeptin 2 h prior to tissue dissection. (G, H) Cell viability of primary hepatocytes isolated from Ctr and L2AKO mice 24 h after treatment with the indicated concentrations of lactacystin alone (G) or in combination with leupeptin (H), *n* = 3. All values are expressed as mean ± SEM. Differences are significant for *P < 0.05, aaccumulation of p62, and ***P < 0.001.

We next assessed the status of the ubiquitin-proteasome system (UPS) that has previously also shown interdependence with CMA *in vitro* (Koga *et al*., 2011b). Using ubiquitin linkage-specific antibodies, we found that part of the reduction in total levels of ubiquitinated proteins in L2AKO mice (Fig.2A) was due to lower levels of K48-linked ubiquitinated proteins, which are classical proteasome substrates (Fig.3A). This reduction was for the most part due to enhanced degradation of ubiquitinated substrates via the proteasome, as *ex vivo* flux analysis using proteasomal inhibitors revealed an increase in their turnover in L2AKO livers (Fig.3B). Despite these increased rates of substrate degradation, the catalytic activities of the proteasome were comparable in Ctr and L2AKO livers (trypsin- and chymotrypsin-like proteasomal activities shown in Fig.3C). Immunoblot analysis of a subset of catalytic and regulatory subunits that comprise proteasomal particles revealed subunit-specific differences in levels of proteasomal proteins between Ctr and L2AKO animals, whereby specific catalytic core alpha subunits, but not beta, and components of the regulatory particle (19S) were more abundant in L2AKO mice (Fig.3D,E). The increase in 19S subunits in L2AKO livers may explain the enhanced degradation of endogenous polyubiquitinated proteins (Fig.3B) which depend on the 19S regulatory particle for access to the core protease, but not of the synthetic peptides used for analysis of catalytic activities that occurs independently of the 19S subunits. As previous studies have shown that subunits of the 20S proteasome can be CMA substrates (Cuervo *et al*., 1995b), we hypothesized that failure to eliminate certain proteasomal components at lysosomes may contribute to their observed higher abundance in L2AKO mice. *In vivo* blockage of lysosomal degradation through injection of leupeptin confirmed that two subunits of the 20S core particle α ring (α3 and α5) undergo degradation in lysosomes from Ctr mice but not from L2AKO mice (Fig.3F). Lastly, experiments in isolated hepatocytes revealed a higher sensitivity of L2AKO cells to proteasome inhibitors (Fig.3G), supporting their higher dependence on intact UPS function and confirming that the observed UPS upregulation in CMA-deficient cells was indeed of compensatory nature. The UPS and other lysosomal pathways, likely macroautophagy, may act cooperatively in this compensation because L2AKO cells revealed higher sensitivity than Ctr cells to simultaneous partial inhibition of both proteolytic systems (Fig.3H).

Together, these data suggest that young L2AKO mice rely on other proteolytic pathways, such as macroautophagy and the UPS, to preserve quality control in the context of CMA failure.

### Hepatic loss of CMA increases cellular susceptibility to proteotoxic and lipotoxic stress

**Figure 4:**
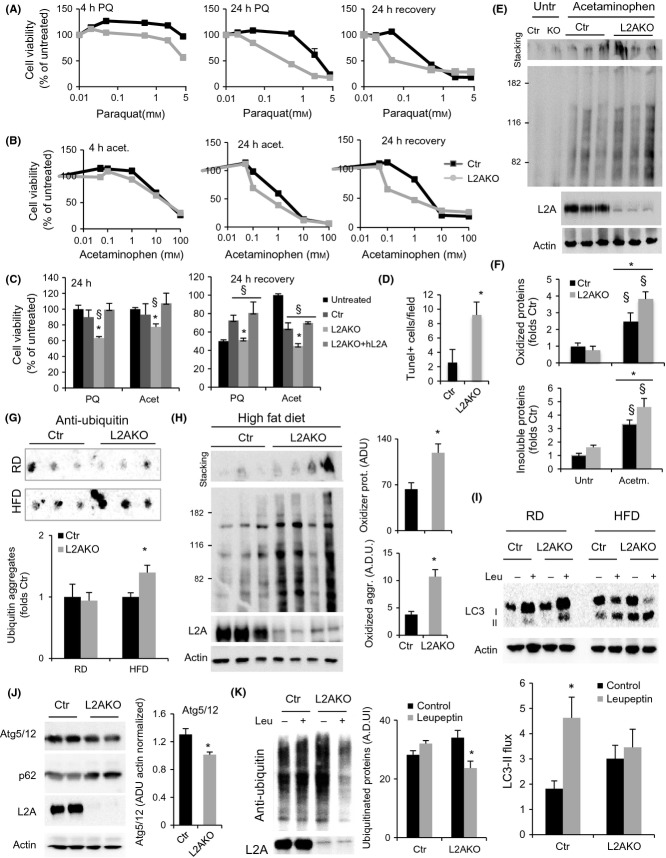
Mice with defective hepatic CMA show loss of proteostasis and increased sensitivity to stress. (A) Viability of primary hepatocytes from 4-month-old control (Ctr) and Albumin-Cre:L2A^f/f^ (L2AKO) mice assessed at 4 or 24 h of exposure to increasing concentrations of paraquat (A) or acetaminophen (B), and 24 h after the stressors was removed, *n* = 3. (C) Viability of L2AKO-deficient cells transfected with an empty vector (L2AKO) or a vector expressing human L2A (+hL2A) upon treatment as indicated, *n* = 6. (D) Quantification of the number of TUNEL-positive cells per section of liver from Ctr and L2AKO mice 24 h after i.p. injection of acetaminophen (representative images are shown in Fig. S3C), *n* = 3. (E) Oxyblot of liver homogenates from Ctr and L2AKO livers treated or not with acetaminophen. Insoluble oxidized proteins are retained in the stacking gel. (F) Densitometric quantification of blots as the one shown in E, *n* = 3. (G,H) Filter retardation assay to assess aggregated poly-ubiquitinated proteins (G) and oxyblot analysis to detect soluble and aggregated oxidized proteins (H) in livers from Ctr and L2AKO mice maintained on a regular chow (RD,*n* = 9 in G, *n* = 6 in H) or a high-fat diet (HFD,*n* = 5 in G, *n* = 6 in H) for 16 weeks. (I) IB of liver homogenates from Ctr or L2AKO mice treated or not with leupeptin 2 h prior to tissue dissection in the same group of animals as in E. Bottom: densitometric quantification, *n* = 3. (J,K) IB for the indicated proteins in liver homogenate from Ctr and L2AKO mice maintained on a HFD for 16 weeks. Right: densitometric quantification of Atg5 (*n* = 6) and ubiquitinated proteins (*n* = 3). All values are expressed as mean ± SEM. Differences are significant for *P < 0.05 compared to Ctr and for § *p* < 0.05 compared to untreated.

Previous studies in cultured fibroblasts have shown that the upregulation of macroautophagy upon partial blockage of CMA (L2A knockdown), similar to that we observed in L2AKO mice livers, is sufficient to maintain cellular homeostasis under basal conditions but that CMA-deficient cells are still more vulnerable to stressors (Massey *et al*., 2006). To determine whether the upregulation of macroautophagy and UPS observed in L2AKO mice livers is sufficient to protect them during different types of stress, we induced oxidative damage in primary hepatocytes with two pro-oxidant agents, paraquat (PQ) and hydrogen peroxide (H_2_O_2_). Analysis of cellular viability right after 4 or 24 h of exposure to the stressors or at 24 h after the stressor was removed (recovery) revealed that L2AKO hepatocytes were more susceptible to oxidative stress (Figs4A and S3A).

Next, we tested whether loss of CMA influences the susceptibility of the liver to hepatotoxic agents such as acetaminophen (Amador-Noguez *et al*., 2007). While viability right after exposure to acetaminophen was comparable in Ctr and L2AKO hepatocytes, CMA deficiency resulted in enhanced cell loss during the recovery after the drug-induced injury (Fig.4B), supporting a role for CMA in restoring cellular homeostasis after drug-induced damage. Transfection of L2AKO cells with a plasmid expressing L2A was sufficient to restore their resistance to both paraquat and acetaminophen (Fig.4C), but this intervention was no longer effective at higher concentrations of the stressor that Ctr and L2AKO cells showed similar sensitivity (Fig. S3C).

To confirm the enhanced vulnerability to stressors in the absence of CMA *in vivo*, we injected Ctr and L2AKO mice with acetaminophen and harvested their livers after 24 h. Staining of liver sections revealed higher number of TUNEL-positive cells in L2AKO mice (Figs4D and S3C), and immunoblot for carbonyl groups demonstrated a trend toward increased oxidized proteins and oxidized proteinaceous aggregates (retained in the stacking gel) in CMA-compromised mice livers (Fig.4E,F). We propose that the diminished resistance of L2AKO mice to hepatotoxic-inducing drugs results, at least in part, from their inability to selectively eliminate damaged proteins under these conditions.

Collectively, these data indicate that although other protein quality control pathways can compensate for loss of CMA, their functions are not redundant as challenging cells with proteotoxic agents and drugs that alter organelle homeostasis elicits their dependence on intact CMA activity.

Next, we tested whether lipotoxic challenges can also expose the vulnerability of CMA-deficient cells. L2AKO hepatocytes in culture displayed a more pronounced decrease in viability than Ctr cells following exposure to increasing concentrations of the free fatty acid, oleate (Fig. S3D). Similarly, a dietary lipid challenge *in vivo* was sufficient to unveil a diminished ability of L2AKO mice livers to sustain hepatic proteostasis. Thus, while L2AKO mice on a regular chow diet (RD) did not exhibit significant differences with Ctr mice in levels of ubiquitinated protein aggregates (Fig. S4A), when we subjected mice to a high-fat diet (HFD) for 16 weeks, CMA-deficient animals displayed a significant increase in ubiquitin-containing aggregates compared to Ctr mice (Fig.4G). Similarly, we observed minimal differences in basal levels of oxidized proteins between Ctr and L2AKO mice on a RD (Fig. S4B), but when subjected to HFD, L2AKO livers had significantly higher content of both soluble and aggregated oxidized proteins (Fig.4H).

We attribute the emergence of altered protein homeostasis in CMA-deficient mice to the fact that dietary lipids compromised the ability of L2AKO mice to compensate for loss of CMA. Thus, whereas L2AKO mice on RD exhibited increased LC3-II flux, indicative of higher autophagic activity, L2AKO mice on a HFD no longer demonstrated the same enhancement of autophagic flux (Fig.4I). HFD L2AKO livers also displayed decreased levels of Atg5/12, accumulation of p62 (Fig.4J) and decreased turnover of ubiquitinated proteins in lysosomes (Fig.4K), all pointing to reduced degradation via macroautophagy under these conditions.

These data support that under basal conditions, blockage of hepatic CMA does not result in pronounced deficits in proteostasis due to active compensatory upregulation of other proteolytic pathways. However, circumstances that diminish the activity of these systems (i.e., diet-induced obesity or chemical inhibition) unmask the vulnerability of CMA-deficient mice and lead to defective protein quality control and accumulation of aggregated and oxidized proteins.

### Aged mice lacking hepatic CMA exhibite liver dysfunction and accelerated metabolic dysregulation

**Figure 5:**
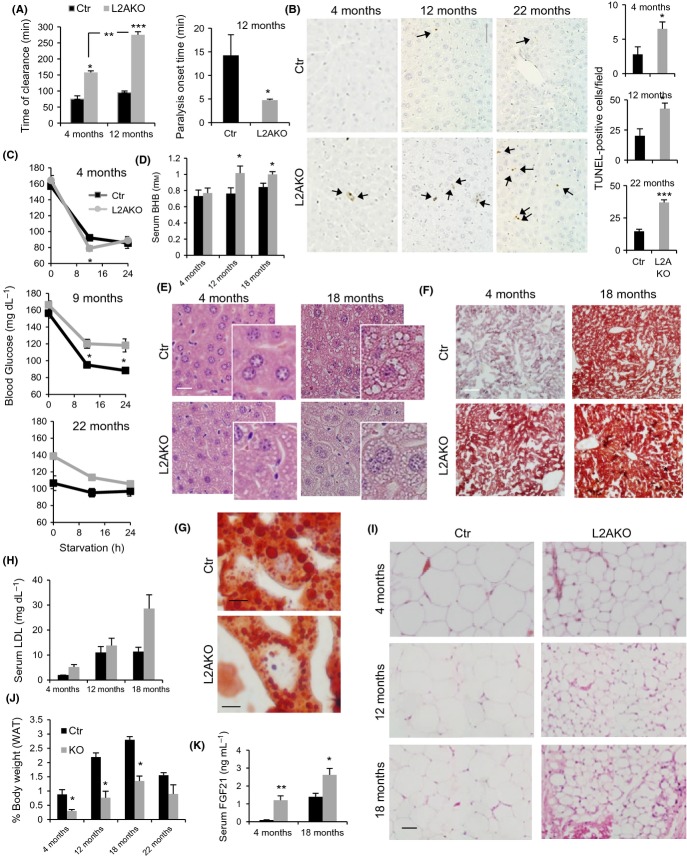
Metabolic changes in CMA-deficient mice with age. (A) Time of clearance (left) and onset of paralysis (right) measured in 4- and 12-month-old control (Ctr) and Albumin-Cre:L2A^f/f^ (L2AKO) mice after zoxazolamine i.p. injection, *n* = 4. (B) TUNEL staining of livers from 4-, 12-, and 22-month-old Ctr and L2AKO mice, scale bar 60 μm (1/4 of full field is shown to appreciate details). Arrows indicate TUNEL-positive cells. Right: quantification of 10 different non-overlapping fields, *n* = 3. (C) Basal and fasting blood glucose levels in 4-, 9-, and 22-month-old Ctr and L2AKO mice, *n* = 3–6. (D) Serum ketone bodies (beta-hydroxybutyrate) in 24 h starved Ctr and L2AKO mice at indicated ages, *n* = 4–8. (E,F) Livers from 24 h starved Ctr and L2AKO mice at the indicated ages were stained with H&E (E) or oil red O (ORO) (F) (scale bar, 40 μm). (G) Magnification of ORO-stained 18-month-old Ctr and L2AKO livers to show lipid droplet size. (H) Serum LDL in 24 h starved Ctr and L2AKO mice at indicated ages, *n* = 4–8. (I) H&E of perigonadal white adipose tissue (WAT) from 24 h starved mice of the indicated ages (scale bar, 100 μm). (J) WAT weight from the same animals, *n* = 3–4. (K) Serum FGF21 levels in 24 h starved Ctr and L2AKO mice, *n* = 5–8. All values are expressed as mean ± SEM. Differences are significant for *P < 0.05; **P < 0.01, and ***P < 0.001.

In light of the susceptibility of L2AKO mice to stress-induced loss of protein homeostasis, we set out to comparatively analyze the impact of aging on liver function, metabolic regulation, and proteostasis in Ctr and CMA-defective animals. To assess changes in hepatic function, we performed a drug clearance test that measures the time needed to metabolize the paralytic agent, zoxazolamine, a drug exclusively cleared by the liver (Amador-Noguez *et al*., 2007; Chen *et al*., 2012). As we have recently reported (Schneider *et al*., 2014), young (4 month) L2AKO mice required significantly longer times for drug clearance when compared with Ctr mice, but differences between Ctr and L2AKO mice were even more pronounced at 12 month of age (Fig.5A). Furthermore, this older group of L2AKO mice displayed a significantly shorter onset of paralysis, measured by the time between treatment and paralysis (Fig.5A). Livers from 12- and 22-month-old L2AKO mice also exhibited a significant increase in TUNEL-positive cells under basal conditions (no stress) compared to age-matched Ctr mice (Fig.5B). Overall, we conclude that aging exacerbates the functional defect of L2AKO mice and reduces hepatocyte viability.

Considering the novel role recently uncovered for CMA in regulating hepatic lipid and glucose metabolism described in young L2AKO mice (Schneider *et al*., 2014), we next analyzed the effect of aging on this metabolic phenotype. Analysis of glycemic control revealed that young L2AKO mice exhibited the previously observed decrease in fasting blood glucose levels attributable to their enhanced hepatic glycolysis (Schneider *et al*., 2014), whereas as L2AKO mice age, they display instead higher fasting blood glucose than age-matched Ctr mice (Fig.5C), implying a degree of glucose intolerance with age. Ketone body production was also significantly higher in 12- and 24-month-old L2AKO mice (Fig.5D), further supporting dysregulated glycemic control in aged mice with impaired hepatic CMA activity.

Changes in lipid metabolism with age in L2AKO mice resembled an accelerated version of those observed during physiological aging in the control group. The high lipid droplet (LD) content and hepatosteatosis initially only observed in young L2AKO augmented progressively with age in this group, but differences with control become less pronounced as lipid accumulated also in the older Ctr mice livers (Fig.5E,F). Interestingly, oil red O staining revealed morphological differences between hepatic LDs in aged Ctr and L2AKO mice, as the LD in L2AKO livers was noticeably smaller and more numerous compared to the larger LDs in Ctr mice (Figs5G and S5A). This gradual worsening of hepatosteatosis with age was associated with significantly higher serum levels of total cholesterol and LDL upon fasting in old L2AKO mice (Figs5H and S5B). Aging reduced the differences between Ctr and L2AKO mice in circulating HDL and serum triglycerides (TG), as the initial reduction in TG observed in young L2AKO mice was reverted back to levels that were no longer significantly different from control (Fig. S5B). We also examined how these changes in serum metabolites in L2AKO mice with age impacted peripheral perigonadal white adipose tissue (WAT). Similar to the effect observed in young L2AKO, aged L2AKO animals exhibited lower WAT weight and smaller adipocyte size than age-matched Ctr mice, but differences between both groups decreased as the oldest Ctr mice group displayed changes in the same direction as the L2AKO mice (Fig.5C,I,J). H&E staining of WAT also revealed an age-dependent increase in infiltration of adipose tissue (hypercellular and fibrotic-like appearance) that was strikingly more evident in aged L2AKO mice (Fig.5I). To start elucidating the possible mechanisms by which the livers of L2AKO mice may influence peripheral fat tissues, we monitored changes in serum of the fibroblast growth factor 21 (FGF21), a protein secreted by the liver that stimulates fat catabolism (Kharitonenkov *et al*., 2005). We observed a significant increase in the levels of FGF21 in the bloodstream in both young and old L2AKO mice compared to Ctr (Fig.5K), suggesting that changes in its hepatic secretion upon CMA blockage could mediate the effect observed in peripheral fat stores. Overall, young L2AKO mice mimicked old Ctr mice in their hepatic lipid accumulation (Fig.5F), reduced size of peripheral fat tissues (Fig.5I), and increased circulating levels of LDL (Fig.5H) and FGF21 (Fig.5K). These findings support that young L2AKO mice phenocopy several of the metabolic alterations that occur in normal aging. Collectively, these data indicate that reduced hepatic CMA activity accelerates the decline in liver function with age and exacerbates the metabolic dysregulation associated with aging.

### Proteostasis failure occurs with age in CMA-deficient livers

**Figure 6:**
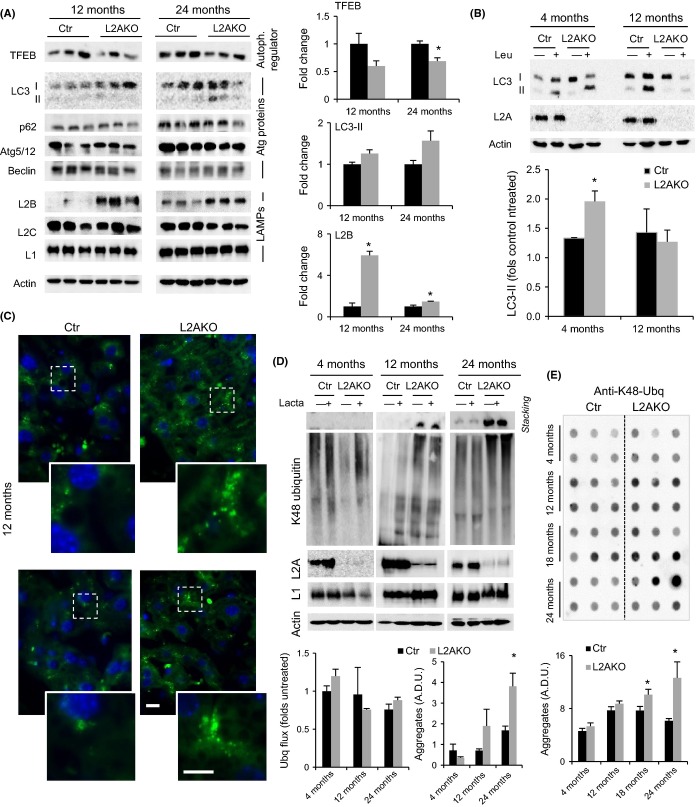
Loss of proteostasis in CMA-deficient mice with age. (A) Immunoblot (IB) of livers from 12- and 24-month-old control (Ctr) and Albumin-Cre:L2A^f/f^ (L2AKO) mice. Right: densitometric quantification, *n* = 3. (B) LC3 flux in livers from 24 h Ctr and L2AKO mice treated or not with leupeptin 2 h prior to tissue dissection. Bottom: densitometric quantification, *n* = 3. (C) Autofluorescence in liver sections from 12-month-old Ctr and L2AKO mouse livers. Nuclei are highlighted with DAPI. Insets: higher magnification of boxed regions. Scale bar 10 μm main. (D) IB for ubiquitinated proteins of livers from 24 h starved Ctr and L2AKO mice incubated in the presence or absence of lactacystin. Insoluble proteins are shown in the gel stacking. Bottom: densitometric quantification, *n* = 3. (E) Filter retardation assay and IB for K48-ubiquitinated proteins from liver homogenates from Ctr and L2AKO mice at the indicated ages. Bottom: quantification of aggregates, *n* = 6. A.D.U. stands for arbitrary densitometric units. All values are expressed as mean ± SEM. Differences are significant for *P < 0.05.

Although unchallenged young L2AKO mice did not reveal protein alterations (oxidation, aggregation, etc.) indicative of altered proteostatic ability (Fig. S4), we postulated that chronic accumulation of hepatic lipids with age in L2AKO mice may ultimately impact the compensatory activity of the other proteolytic systems, similar to the effect observed in mice subjected to a HFD (Fig.4). Analysis of various autophagy markers in older Ctr and L2AKO mice livers revealed that, contrary to the upregulation of TFEB observed in young L2AKO mice (Fig.2E-G), levels of TFEB were diminished compared to Ctr in 12- and 24-month-old L2AKO mice (Fig.6A) as was also its nuclear localization (Fig. S6A). Similarly, the increase in LAMP2-B levels, still observed at 12 month of age in L2AKO mice, was markedly reduced in the oldest L2AKO mice group (Fig.6A). Consistent with this reduction in positive regulators and effectors of macroautophagy in L2AKO with age, we observed a gradual accumulation of basal LC3-II with age in L2AKO animals compared to age-matched Ctr (Fig.6A). Lastly, we confirmed that the increase in LC3 flux observed in young L2AKO was no longer evident in 12-month-old L2AKO mice, which even displayed a trend toward lower flux than Ctr mice (Fig.6B). We also observed that by 12 month, hepatocytes from L2AKO mice contained more lipofuscin granules than their Ctr counterparts (Figs6C and S6B,C), indicating a premature compromise in the lysosomal system in the context of CMA loss and a possible consequence of reduced macroautophagy (Hohn & Grune, 2013). Together, these data support that macroautophagy compensation in response to CMA blockage is not sustained with age.

Next, considering the enhanced degradation through the proteasome in young L2AKO mice (Fig.3B), we evaluated possible changes in UPS activity in these animals with age. We found that, contrary to the increased proteasomal degradation of K48-linked ubiquitinated proteins in young L2AKO mouse livers, flux through the UPS decreased with age to levels comparable or even lower than age-matched Ctr mice (Fig.6D). This inability to enhance proteasomal degradation may explain the higher abundance of ubiquitin-containing aggregates in L2AKO mice as they age, evidenced as proteins retained in the stacking gel during electrophoresis (Fig.6D) and confirmed through filter retardation assay (Fig.6E).

Together, these studies show that the initial compensatory upregulation of macroautophagy and UPS is not sustained with age and that loss of this compensation accelerates the age-related decline in proteostasis in L2AKO mice.

### CMA failure in aged animals increases vulnerability to oxidative stress

**Figure 7:**
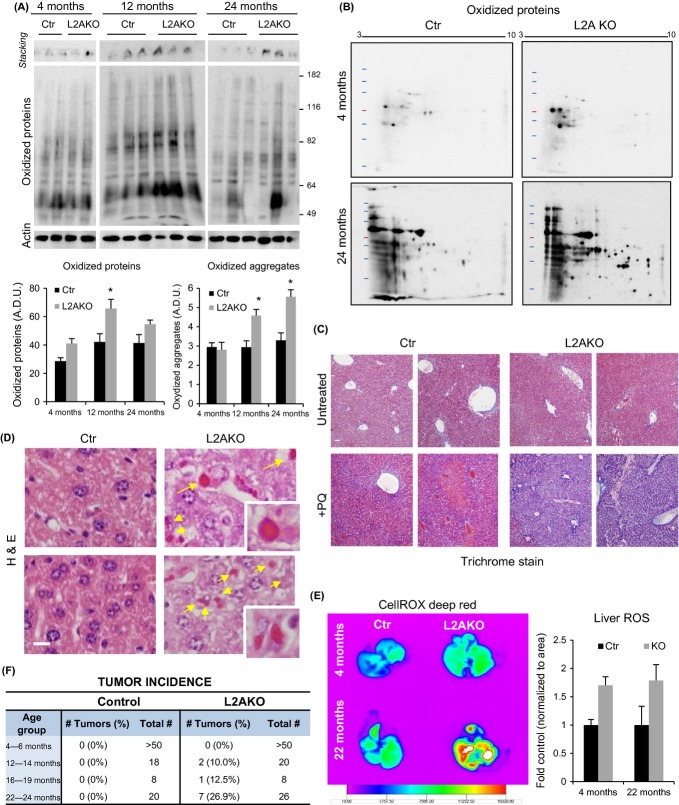
Enhanced susceptibility to oxidative stress of CMA-deficient mice. (A) One-dimensional oxyblot analysis to detect soluble and aggregated oxidized proteins in 4-, 12-, and 24-month-old control (Ctr) and Albumin-Cre:L2A^f/f^ (L2AKO) mice. Bottom: densitometric quantification, *n* = 4–6. (B) Two-dimensional electrophoresis followed by oxyblot analysis in livers from 4- and 24-month-old Ctr and L2AKO mice. (C) Trichrome stain of livers from 22-month-old Ctr and L2AKO mice treated or not with two low-dose injections of paraquat 1 week prior and one high-dose injection of paraquat 24 h prior to tissue collection. (D) H&E stain of the same livers as in (C) to show the appearance of intracellular Mallory bodies (yellow arrows and insets) in L2AKO mice. (E) *Ex vivo* imaging of livers extracted from Ctr and L2AKO mice of the indicated ages treated with paraquat, after injection of a fluorogenic probe for the detection of ROS. Right: quantification of ROS levels, *n* = 3. (F) Tumor incidence in Ctr and L2AKO mice from different age groups. All values are expressed as mean ± SEM. Differences are significant for *P < 0.05.

To further understand how age-related CMA failure and the lack of compensatory response by other proteolytic systems with age impact protein quality control in older organisms, and whether it could led to accumulation of damage, we measured levels of oxidatively modified proteins *in vivo*. We focused on oxidized proteins because of the previously described role for CMA in their turnover (Kiffin *et al*., 2004) and because in this work, we found that loss of CMA increases the susceptibility of primary mouse hepatocytes to oxidative stress (Fig.4A). Compared to age-matched Ctr mice, livers from 12- and 24-month-old L2AKO mice displayed higher amounts of soluble and aggregated (retained in the stacking gel) oxidized proteins (Fig.7A). In addition to these quantitative changes, bi-dimensional electrophoresis also revealed qualitative changes in the subset of the proteome undergoing oxidative damage in the livers from 24-month-old L2AKO mice compared to Ctr mice (Fig.7B).

Next, we treated mice with the pro-oxidant agent paraquat to examine how the age-related decline in proteostasis in CMA-incompetent animals influenced their ability to deal with an oxidative stress challenge *in vivo*. Trichrome staining of liver sections revealed minimal differences between untreated and treated Ctr and L2AKO mice of 4 or 12 month of age (Fig. S7A). However, 22-month-old L2AKO mice displayed an exacerbated hepatic fibrotic reaction upon PQ-induced oxidative stress (Fig.7C) and H&E staining detected the appearance of intracellular inclusions, known as Mallory bodies (Hanada *et al*., 2012) (Fig.7D). These highly eosinophilic structures are typically positive for ubiquitin and are indicative of protein aggregation. To directly compare levels of oxidative stress in Ctr and L2AKO mice *in vivo*, we delivered a fluorogenic probe via tail vein injection that is nonfluorescent while in a reduced state and exhibits fluorescence upon oxidation by reactive oxygen species (Scharf *et al*., 2013). *Ex vivo* radiometric analysis of the livers revealed an increase in the signal in old L2AKO mice compared with Ctr mice, in support of higher oxidative stress in the CMA-incompetent mice (Fig.7E). Unexpectedly, L2AKO mice also displayed higher ROS levels at early ages (Fig.7E), despite the minimal pathological changes observed in these livers (Fig. S7A).

Multiple factors could contribute to the efficient handling of the higher ROS content in young L2AKO mice, including their ability to compensate for loss of CMA by upregulating other proteolytic systems to clear ROS-mediated damage (Fig.3). To further investigate this protection against ROS damage in young L2AKO mice, we analyzed levels of proteins involved in the oxidative stress defense in both groups of animals. We did not find significant differences in hepatic content of SOD1 or catalase (Fig. S8A), and in fact, studies in animals injected with leupeptin did not reveal significant changes in their levels, suggesting that they do not undergo lysosomal degradation (Fig. S8B). In contrast, a proteomic analysis of liver lysosomes isolated from leupeptin-treated young Ctr and L2AKO mice (Schneider *et al*., 2014) identified a group of enzymes involved in the cellular antioxidant response among the subset of hepatic CMA substrate proteins. Figure S8C shows four of the enzymes that undergo lysosomal degradation in livers of Ctr mice but whose degradation is significantly reduced in CMA-incompetent animals. These enzymes all bear CMA targeting motifs in their sequence in further support of their possible degradation by this pathway (Fig. S8D). To determine whether the reduced lysosomal degradation of these antioxidant enzymes in L2AKO mice leads to higher hepatic content in these animals, we used glyoxalase I (Glo 1) as an example (Kim *et al*., 2012). We found that levels of Glo1 were significantly higher in cytosolic fractions from L2AKO mice than in Ctr (Fig. S8E), but Glo1 reached levels similar to those observed in Ctr mice upon induction of oxidative stress by paraquat treatment (Fig. S8F). We propose that higher levels of these proteins due to their reduced degradation through CMA may, in the short run, help with handling of toxic species and by-products. It is likely that the progressive alterations in proteostasis observed in older L2AKO mice eventually compromise homeostasis of these protective proteins and lead to loss of their function and increased susceptibility to hepatic damage (Fig. 7C,E).

We attribute the increasing signs of liver damage and reduced function in aged L2AKO mice to metabolic dysregulation compounded by the gradual decline in proteostasis over time. Both steatosis (Park *et al*., 2010) and reduced quality control (Takamura *et al*., 2011) have been shown to promote oncogenic transformation. Interestingly, we observed increased incidence of hepatic tumors in the older group of L2AKO mice, while no spontaneous hepatic tumors were detected in age-matched Ctr mice throughout the course of this study (Figs7F and S7B). Pathological analysis classified some of the tumors as hepatocellular adenoma with areas of eosinophilic foci (proteinaceous aggregates), lipidosis, and inflammation (Fig. S7C), and immunohistochemistry for L2A confirmed that tumor cells were derived from L2AKO hepatocytes and not from another cell type (Fig. S7D). It is likely that the combined chronic hepatosteatosis, accelerated poor quality control, and increasing oxidative damage with age may facilitate malignant transformation and tumor formation in the CMA-deficient livers.

Overall, these studies support that the age-dependent decline in hepatic CMA activity may contribute to the metabolic dysregulation characteristic of old organisms. The gradual diminished ability of other proteolytic systems to compensate for CMA loss will add alterations in proteostasis to the already metabolically compromised liver, further enhancing liver damage, risk of malignant transformation, and accelerating loss of liver function with age.

## Discussion

In this work, we have found that whereas the loss of the regulatory functions of CMA in liver, such as those involved in the control of carbohydrate and lipid metabolism, cannot be compensated for – leading to overt metabolic phenotypes upon CMA failure –, the contribution of CMA to quality control in young mice can be effectively handled by other proteolytic systems. These compensatory responses, however, do not imply redundancy of the proteolytic systems, as conditions such as stress (dietary lipid challenges, oxidant, and hepatotoxic agents) or aging unmask the reliance of cells on CMA (Fig. S9). We postulate that the gradual decline in protein quality control, reduced resistance to stress, and altered metabolic homeostasis in aging L2AKO mice contributes to hepatocyte dysfunction and may even favor malignant transformation and liver tumorigenesis.

Upregulation of the other proteolytic systems is sufficient to maintain proteostasis in a context of defective CMA under basal conditions, but not in the face of different stressors that would typically drive CMA activity such as proteotoxic, lipotoxic, or hepatotoxic agents *in vivo*. Interestingly, this susceptibility to stress in CMA-deficient cells was mostly manifested during the recovery phase after stress and not necessarily during the stress phase. Dependence on CMA for cellular survival poststress could stem from its ability to selectively remove the subset of CMA substrates damaged during the stress to restore cellular homeostasis. Although both macroautophagy (Scherz-Shouval *et al*., 2007) and CMA (Kiffin *et al*., 2004) become activated during oxidative stress, they are not redundant in their protective functions. Conditions with predominantly protein damage benefit from the selectivity of CMA, whereas organelle injury and accumulation of protein aggregates are handled for the most part by macroautophagy. The enhanced oxidative damage observed in the stressed or old L2AKO mice *in vivo* further supports a physiological role for CMA in the oxidative stress response and suggests that the decline of CMA with age lowers the threshold of oxidative insults that cells can tolerate.

Different mechanisms could contribute to the enhanced activity of macroautophagy and the UPS pathway observed *in vivo* in the CMA-incompetent mice. We present here that the compensatory activation may be partially caused by the accumulation of macroautophagy- and proteasomal-related proteins in L2A-deficient cells. We found that specific subunits of the catalytic and regulatory proteasome as well as key regulators of macroautophagy, such as TFEB, undergo lysosomal degradation in control animals but not in L2A-deficient mice. These findings support that under normal conditions, CMA activity could also contribute to regulating the levels of these, and maybe other, effector components of other proteolytic systems. Upon CMA compromise, the accumulating levels of macroautophagy and proteasomal effectors could lead to the observed enhanced activity of these pathways and allow hepatocytes to maintain proteostasis even in the absence of CMA. We have shown a ‘gain of function’ outcome in young L2AKO mice for other CMA substrates involved in metabolic regulation, such as glycolytic and lipogenic enzymes due to their cytosolic accumulation (Schneider *et al*., 2014). A similar situation could apply to TFEB, the master regulator of the macroautophagy/lysosomal transcriptional program, whose levels were markedly increased in the young L2AKO livers. We demonstrate that the increased abundance of TFEB is in part due to its reduced degradation, although as TFEB regulates its own transcription (Settembre *et al*., 2013), it is likely that part of the increase is also at the transcriptional level. Activation of TFEB requires its dephosphorylation and nuclear translocation. As we observed higher nuclear TFEB levels, we propose that either the high levels of TFEB observed in these animals may overpass the phosphorylating ability of the regulatory kinases, or that yet to be identified TFEB phosphatases may also be elevated in these animals. In the case of the UPS, the previously reported selective degradation of 20S proteasome subunits by CMA (Cuervo *et al*., 1995b) and the CMA-dependent degradation of 19S regulatory components identified in this work could be the main driver for the observed increased proteolytic flux through this system.

The specific reasons behind the reduced compensatory activity of other proteolytic systems for CMA loss with age require further investigation. However, based on the molecular mechanisms identified in this work that contribute to the compensatory activation in young animals, it is possible that persistent lack of selective turnover by CMA of effectors of the macroautophagy and UPS systems may lead to their deregulation. For example, persistence of TFEB molecules in the cytosol of aging L2AKO where proteostasis is already compromised may lead to unwanted modifications and subsequent aggregation, thus explaining the low levels of TFEB levels in these animals, even below those observed in age-matched controls. Compensatory decline could also be due to the factors associated with chronic upregulation of the proteolytic systems such as component exhaustion or even a direct negative impact of the metabolic changes on the proteolytic systems. For example, high intracellular lipid content exerts an inhibitory effect on macroautophagy by altering the dynamics of lysosome and autophagosome fusion (Koga *et al*., 2010).

A similar enhanced function of CMA substrates could be behind the tolerance of young L2AKO mice to the higher levels of ROS detected in these animals. We have identified as *bona fide* CMA substrates a subgroup of proteins involved in the cellular response to oxidative stress. Although the higher levels of these proteins observed in young L2AKO due to their reduced degradation by CMA could help these animals to deal with their higher ROS levels, persistent buildup of some of these glutathione-consuming enzymes may impact the ability of L2AKO mice to neutralize free radicals and peroxides as they age. Efforts should be invested to further elucidate the role of CMA in regulating several of these enzymes under physiological conditions and to understand how their lack of turnover in lysosomes influences the cellular response to oxidative stress.

Regarding the progression with age of the metabolic phenotype in CMA-deficient livers, it is interesting that the striking metabolic differences between young Ctr and L2AKO mice become progressively reduced as the metabolic changes in the old Ctr start resembling those already observed in the L2AKO mice early in life. In this respect, CMA compromise phenocopies some of the metabolic characteristics of an aging liver. Interestingly, the onset of metabolic dysfunction early in life did not coincide with simultaneous loss of protein quality control in L2AKO mouse livers which does not appear until later on. In light of the temporal differences in the onset of metabolic and quality control phenotypes, we propose that chronic accumulation of hepatic lipids may eventually impact proteostasis. For example, even in young L2AKO mice, a dietary lipid challenge was sufficient to cause decompensation of the proteolytic systems and precipitate loss of proteostasis. Moreover, further compromise in proteostasis with age may also negatively impact metabolic regulation, creating a vicious cycle that ultimately results in the age-related functional decline.

## Experimental procedures

### Animals, diets, and treatments

Male C57BL/6 mice (wild-type or transgenic for Albumin-Cre) 3–5 months of age were purchased from Jackson Laboratory. The mouse model with a conditional deletion for L2A in the liver was generated as previously described (Schneider *et al*., 2014). All mice were housed under pathogen-free conditions and handled according to protocols approved by the Institutional Animal Care and Use Committee of Albert Einstein College of Medicine. Treatments and liver functional analyses were done as described before (see Supporting Information).

### Chemicals and antibodies

Sources of chemicals and antibodies were used as described before (Kiffin *et al*., 2004; Zhang & Cuervo, 2008) (see also Supporting Information).

### Histological procedures and electron microscopy

Livers were fixed in 10% neutral-buffered formalin and stained with H&E, Trichrome, or TUNEL, where indicated. For oil-red-O staining, liver tissue was frozen in OCT, sectioned, and stained. For immunohistochemistry, deparaffinized and unstained liver sections were processed using standard procedures for epitope retrieval, quenched, and blocked before incubation with the desired primary antibody. Electron microscopy was done following the standard procedures, and morphometric analysis was performed in micrographs using the standard criteria defined before (Singh *et al*., 2009) (see also Supporting Information).

### Measurement of macroautophagy and proteasome activities

To assess LC3 flux in an *ex vivo* setting, livers were harvested, minced, and incubated at 37 °C in DMEM with or without leupeptin. To measure LC3 flux *in vivo*, mice were treated with an i.p. injection of either saline or leupeptin (20 mg kg^−1^ b.w.) 2 h before tissue harvesting and samples were analyzed by immunoblot for LC3. Catalytic activities of the proteasome were determined as previously described (Amador-Noguez *et al*., 2007) (see also Supporting Information).

### Subcellular fractionation and isolation of lysosomes

Mouse liver lysosomes were isolated from a light mitochondrial–lysosomal fraction in a discontinuous metrizamide density gradient, and a fraction enriched in the subpopulation of lysosomes active for CMA was further separated by differential centrifugation as previously described (Cuervo *et al*., 1997). Lysosomal integrity was verified after isolation by measuring β-hexosaminidase activity latency, and only preparations with <10% broken lysosomes were used (Storrie & Madden, 1990). To obtain cytosolic fractions, the supernatant of the light mitochondrial–lysosomal fraction was subjected to centrifugation at 100 000 *g* for 1 h at 4 °C in a Beckman TL-100 Ultracentrifuge (TLA-100 rotor, Beckman, Jersey City, NJ, USA) and the supernatant was collected as the cytosolic fraction.

### Proteostasis analysis

Levels of oxidized proteins in isolated samples were determined by quantifying the total protein carbonyl content following the manufacturer's instructions for the OxyBlot Oxidized Protein Detection Kit (Chemicon International, Temecula, CA, USA) (see also Supplemental Materials). Protein aggregation was determined using the filter retardation assay (Massey *et al*., 2006) or as material retained in the stacking gel analysis after subjecting samples to conventional SDS–PAGE (see also Supporting Information).

### *In vivo* measurement of reactive oxygen species

Levels of hepatic ROS were measured using the fluorogenic probe CellROX Deep Red Reagent C (Molecular Probes, Eugene, OR, USA) as described before (Scharf *et al*., 2013). Briefly, anesthetized mice were tail-vein-injected with 50 μL (2.5 mm) of the fluorescent probe, and after 20 min, the organs were resected for imaging in the *In Vivo* Imaging System (IVIS, Kodak Image Station 400MM PRO, Carestream Health, New Haven, CT, USA).

### Quantification of serum metabolites

Blood glucose levels were monitored at the indicated times using blood collected from tail veins and a glucometer (Accu-Chek Compact; Roche, Pleasanton, CA, USA). For analysis of metabolites in serum, blood was collected by retro-orbital bleed, serum was separated by centrifugation, and FFA (HR Series NEFA, Wako Diagnostics Mountain View, CA, USA), TG (Infinity Triglyceride, Thermoscientific, Springfield Township, NJ, USA), and ketone (β-Hydroxybutyrate LiquiColor® Reagent, Stanbio, Boerne, TX, USA) levels were determined by colorimetric assays. Cholesterol, HDL, and LDL levels were measured by the Biomarker Analytical Research Core at the Albert Einstein College of Medicine. Serum FGF21 levels were measured using specific ELISA (Biovendor, Brno, Czech Republic).

### Other methods

Cell viability, protein concentration, immunoblot, and image analysis were performed following standard procedures (see Supporting Information).

### Statistical analysis

All numerical results are reported as mean + standard error of the mean (SEM), and statistical significance was determined using the two-tailed unpaired Student's *t*-test (single comparisons) or ANOVA followed by the Bonferroni *post hoc* test (multiple comparisons).

## Supporting Information

**Fig. S1** Lysosomal markers in Albumin-Cre-L2A^f/f^ mice. **Fig. S2** Compensation by macroautophagy for loss of CMA activity in liver. **Fig. S3** Response of young CMA-deficient mice to different stressors. **Fig. S4** Proteostasis in young CMA-deficient mice. **Fig. S5** Metabolic changes in aged CMA-defective mice with age. **Fig. S6** Failure of macroautophagy compensation and decreased proteostasis in old mice with compromised CMA. **Fig. S7** Increased vulnerability of CMA-impaired animals to oxidative stress and malignant transformation. **Fig. S8** Proteins involved in the cellular oxidative stress response in livers of CMA-impaired animals. **Fig. S9** Model of the age-related changes in dual role of CMA in maintaining metabolic homeostasis and protein quality control. **Data S1** Expanded experimental procedures. **Data S2** Additional references.
