# Supplementary material for: Loss of hepatic chaperone-mediated autophagy accelerates proteostasis failure in aging
Source: Aging Cell. 2015 Jan 23;14(2):249–64. doi: 10.1111/acel.12310 (PMC4364837; doi:10.1111/acel.12310)
Supplement: Supplementary file 1 [file acel0014-0249-sd1.pdf]

# Loss of hepatic chaperone-mediated autophagy accelerates proteostasis failure in aging

Jaime L. Schneider[1,2], Joan Villarroya[1,2,5], Antonio Diaz-Carretero[1,2], Bindi Patel[1,2], Aleksandra Urbanska[3], Mia M. Thi[4], Francesc Villarroya[5], Laura Santambrogio[3], and Ana Maria Cuervo*[1,2]

## Supporting Information Listing

Supplementary figures 1-9 including legends

Expanded experimental procedures

Additional references

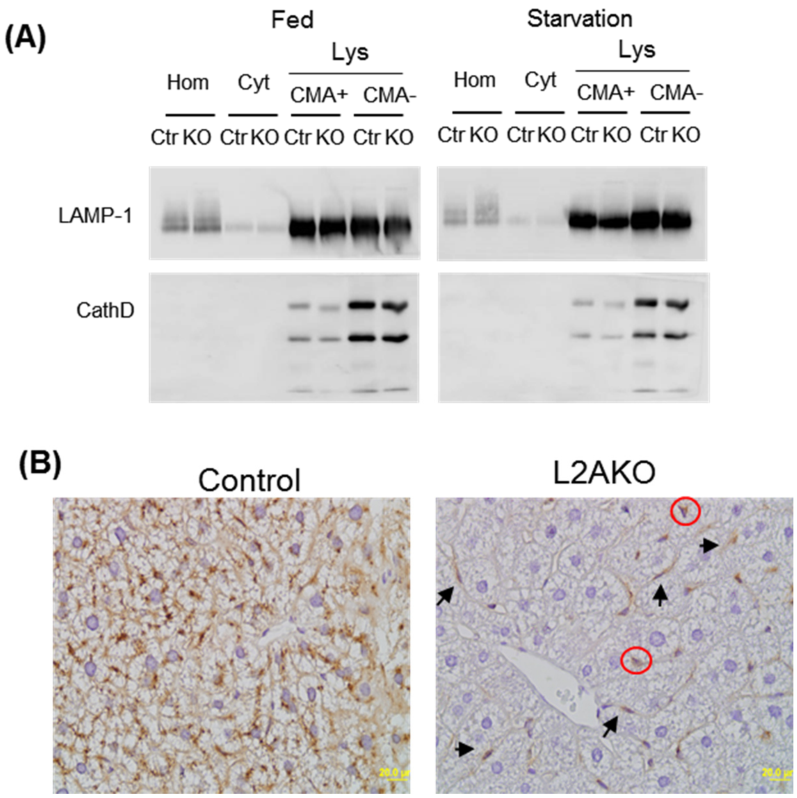


**Supplementary Figure 1. Lysosomal markers in Albumin-Cre-L2A$^{f/f}$ mice. (A)** Immunoblot (IB) of homogenate (Hom), cytosol (Cyt), and lysosomes (Lys) with high (+) or low (-) CMA activity isolated from livers of fed or 24h starved control (Ctr) or Albumin-Cre:L2A$^{f/f}$ (L2AKO) mice. These fractions are from the same subcellular fractionation shown in Fig. 1A. **(B)** Immunohistochemistry for LAMP-2A in liver sections from the same mice. Examples of liver cell types other than hepatocytes are marked with arrows (endothelial cells) or circles (Kupfer cells). (scale bar, 20 $\mu$m).

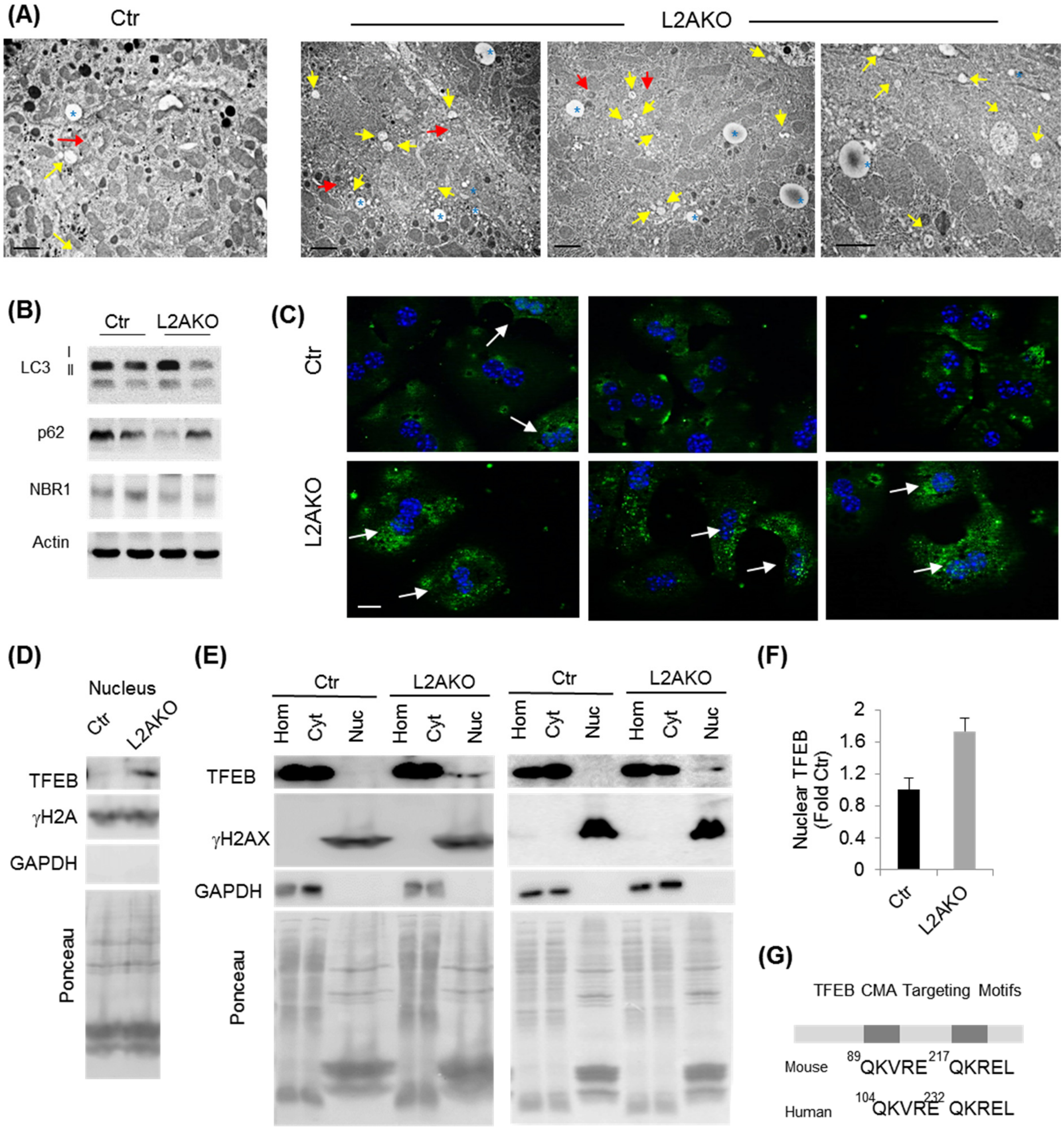


**Supplementary Figure 2. Compensation by macroautophagy for loss of CMA activity in liver.** **(A)** Electron microscopy images from liver of control (Ctr) and L2AKO mice. Arrows indicate examples of autophagosomes (red) or autophagolysosomes (yellow). Blue stars mark lipid droplets). (scale bar, 5 μm). **(B)** IB for macroautophagy markers in livers of 24h starved Ctr and L2AKO mice. **(C)** Immunofluorescence for TFEB in hepatocytes from Ctr and L2AKO mice. Arrows indicate cells with nuclear TFEB signal. (scale bar, 10 μm). **(D-F)** IB of nuclear fractions from livers of Ctr and L2AKO mice using a NP-40 based lysis method (D) or the NE-PER lysis Kit (Pierce) (two independent preparations are shown) (E). γH2AX and GAPDH are shown as nuclear and cytosolic markers, respectively and staining with Ponceau is used as loading control. Densitometric quantification of blots as the ones shown in D and E is shown in F (n=6). **(G)** Scheme of the CMA targeting motifs in mouse and human TFEB.

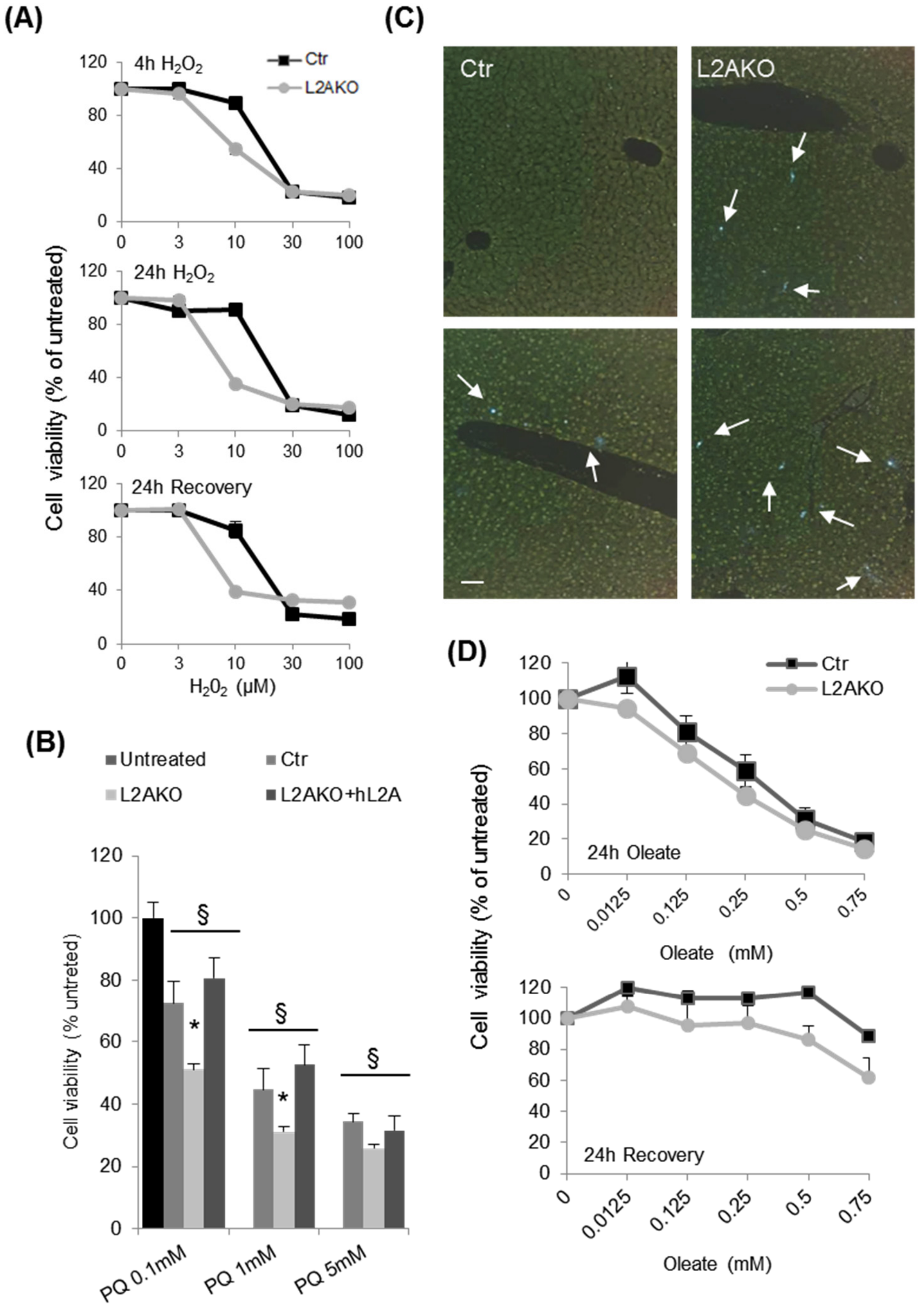


**Supplementary Figure 3. Response of young CMA-deficient mice to different stressors. (A,D)** Viability of primary hepatocytes isolated from control (Ctr) and Albumin-Cre:L2A$^{f/f}$ (L2AKO) mice assessed right after 4h or 24h of exposure to increasing concentrations of hydrogen peroxide **(A)** or oleate **(D)** and 24h after the stressor was removed, n=3. **(B)** Viability of cells Ctr, L2AKO or L2AKO transfected with a plasmid expressing human L2A (hL2A) 24h after addition of the indicated concentrations of paraquat (PQ), n=6. **(C)** TUNEL staining of liver sections from Ctr and L2AKO mice 24h after i.p. injection of acetaminophen. Arrows indicate TUNEL positive cells. (two different fields shown, scale bar: 100µm). Values are expressed as mean±s.e.m. Differences with Ctr (*) or with untreated cells (§) are significant for $p<0.05$

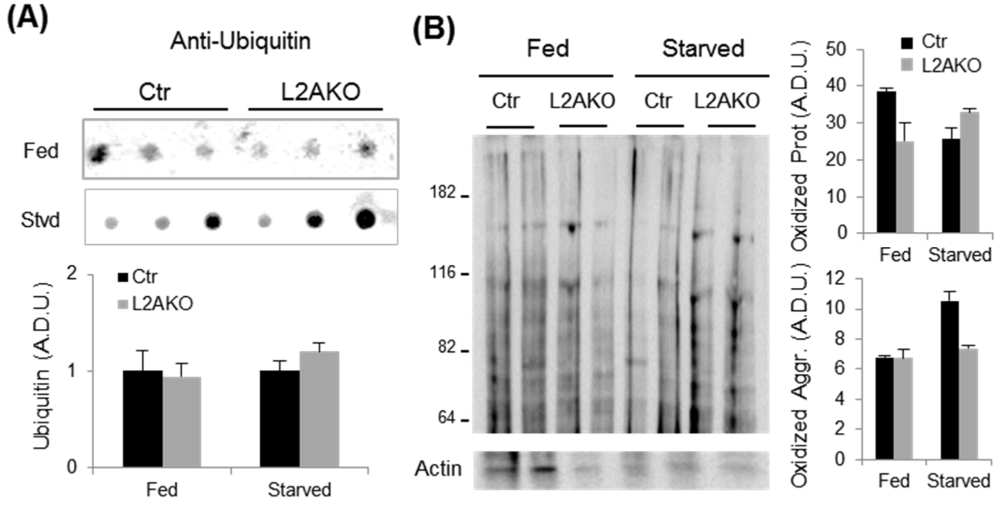


**Supplementary Figure 4. Proteostasis in young CMA-deficient mice.** Filter retardation assay for poly-ubiqiutinated proteins **(A)** and oxyblot analysis **(B)** in livers from Ctr and L2AKO mice fed (n=9 in **A**, n=2 in **B**) or starved for 24h (n=12 in **A**, n=2 in **A**). Desitometric quantifications are shown at the bottom and right for A and B, respectively. Values are expressed as mean±s.e.m.

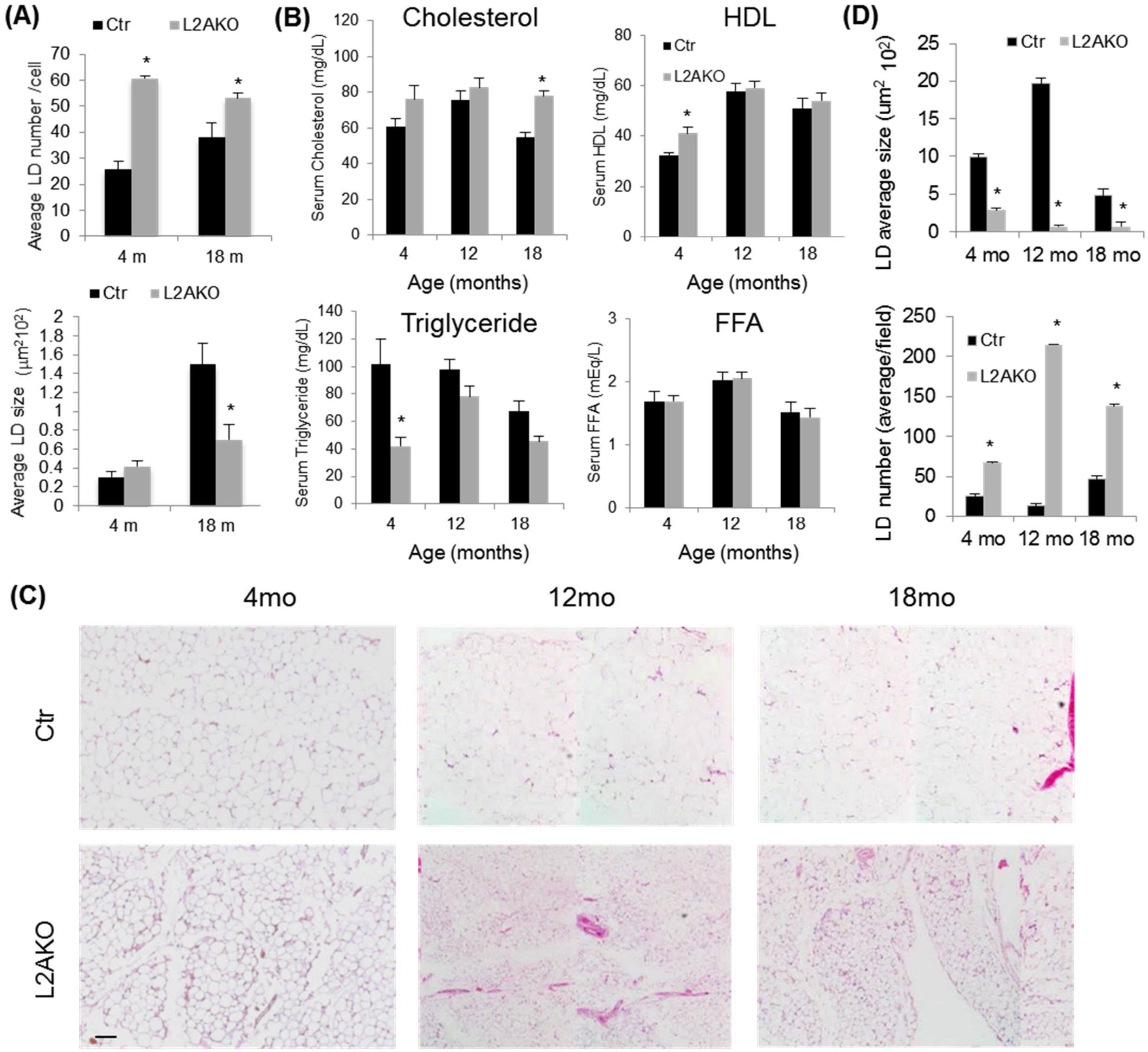


**Supplementary Figure 5. Metabolic changes in aged CMA-defective mice with age. (A)** Average number (top) and size (bottom) of lipid droplets (LD) in livers of control (Ctr) and Albumin-Cre:L2A$^{f/f}$ (L2AKO) mice of the indicated ages stained with oil-o-red (representative images shown in Fig. 5F,G), n=4-8. **(B)** Levels of the indicated lipids in serum collected by retro-orbital puncture from 24h starved control (Ctr) and Albumin-Cre:L2A$^{f/f}$ (L2AKO) mice of the indicated ages, n=4-8. **(C)** H&E of perigonadal white adipose tissue (WAT) harvested from 24h starved Ctr and L2AKO mice of the indicated ages, (scale bar: 100μm). **(D)** Quantification of the average size and number of lipid droplets (LD) in images as the ones shown in C, n =4. Values are expressed as mean±s.e.m. Differences are significant for * $p<0.001$

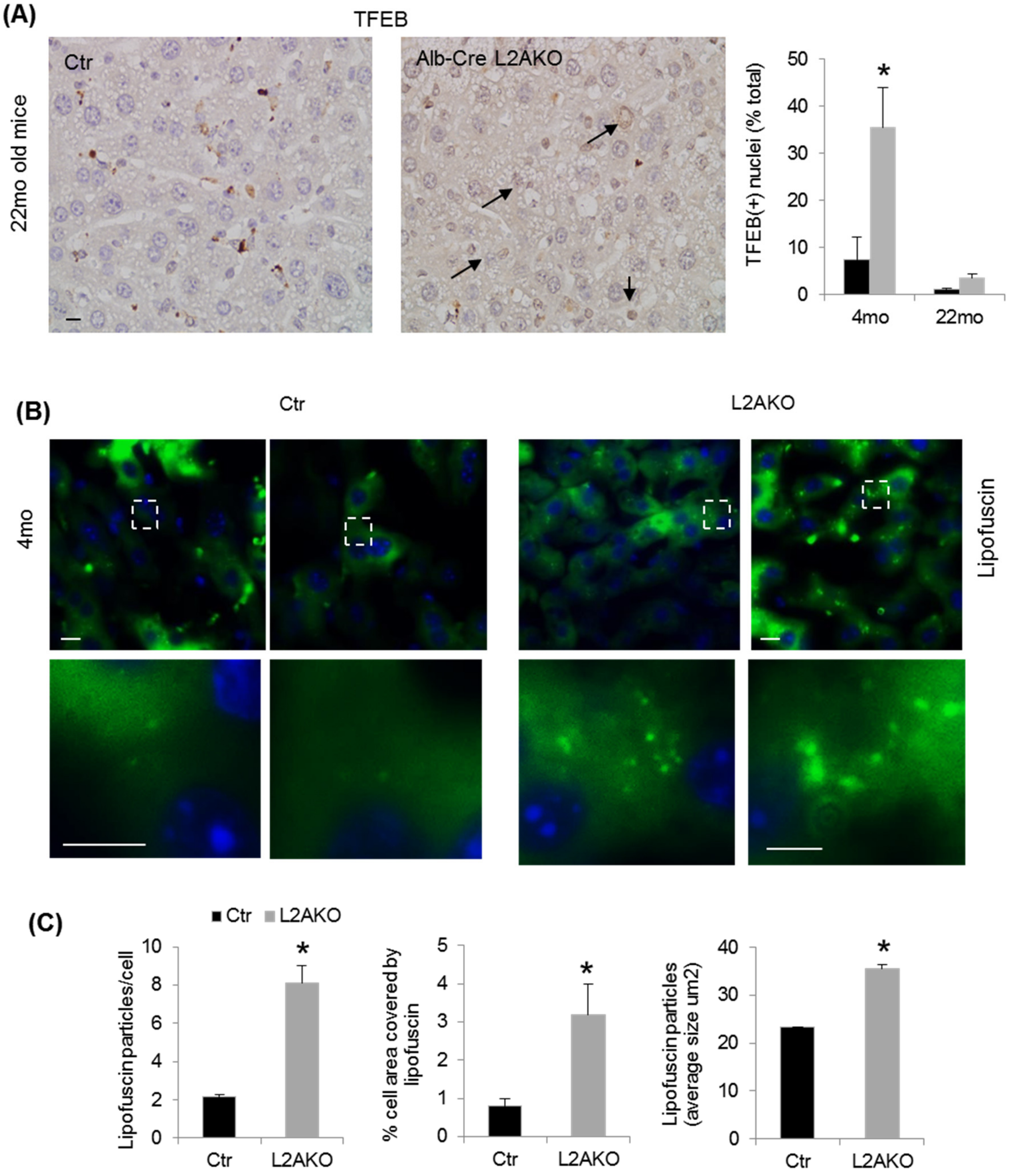


**Supplementary Figure 6. Failure of macroautophagy compensation and decreased proteostasis in old mice with compromised CMA. (A)** Immunohistochemistry for TFEB of liver sections from 22 months old control (Ctr) and Albumin-Cre:L2A$^{f/f}$ (L2AKO) mice starved for 24h. Black arrows: nuclear staining, (scale bar: 10μm). Right: Quantification of cells with nuclear staining for TFEB obtained from the micrographies shown here for 22mo and in main Fig. 2 for 4 mo, n=4. **(B)** Representative images showing lipofuscin autofluorescence in 4mo old Ctr and L2AKO mice. Insets show boxed areas at higher magnification, (scale bar: 10μm). (**C**) Quantification of the number, average size and percentage of cellular area covered by lipofuscin particles calculated from images as the ones shown here and in main Fig. 6. n = 4 Values are expressed as mean$\pm$s.e.m. Differences are significant for * $p<0.01$.

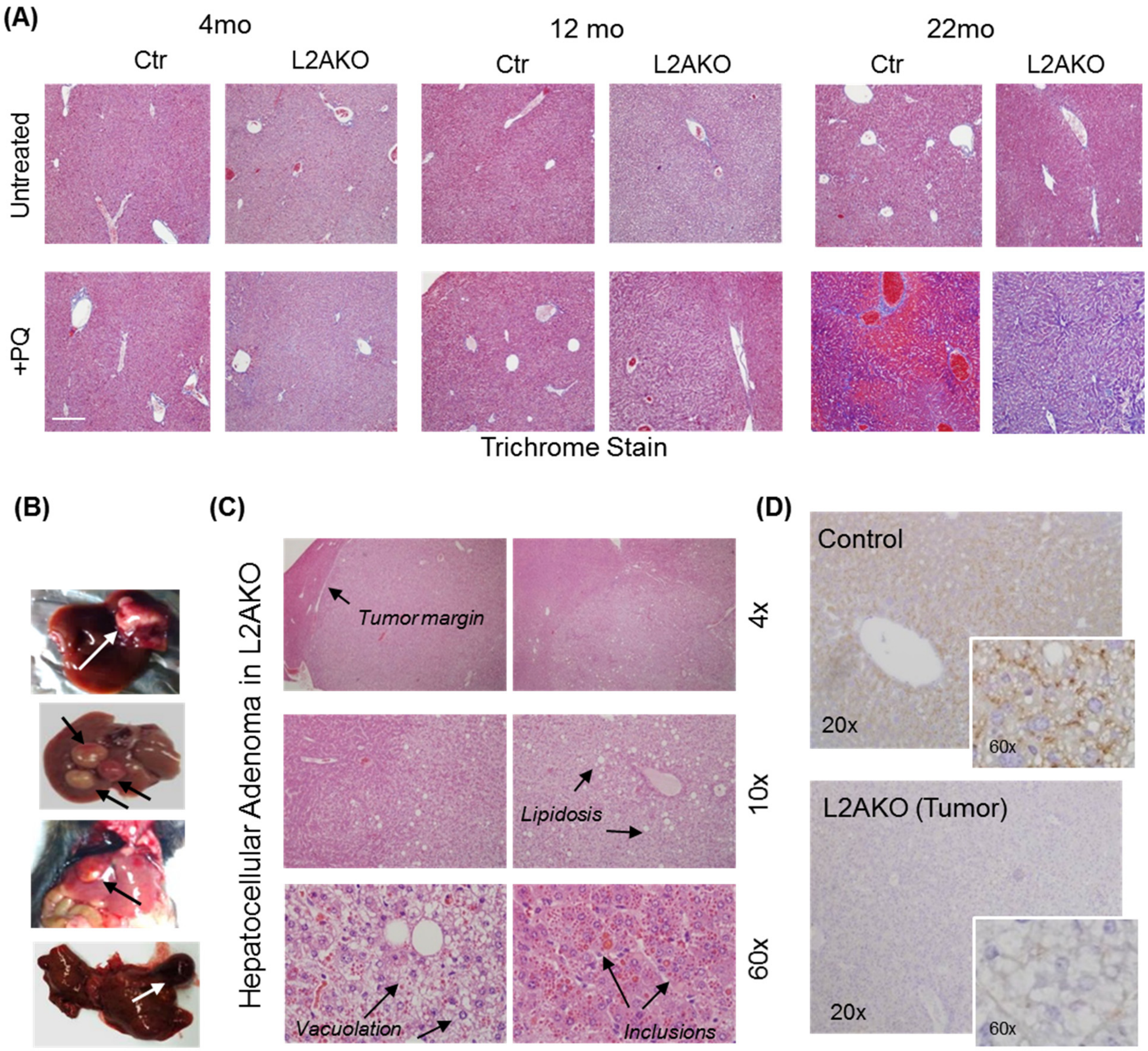


**Supplementary Figure 7. Increased vulnerability of CMA-impaired animals to oxidative stress and malignant transformation.** **(A)** Trichrome stain of livers from control (Ctr) and Albumin-Cre:L2A$^{f/f}$ (L2AKO) mice of the indicated ages treated or not with two low-dose injections of paraquat one week prior and one high-dose 24h prior to tissue collection (scale bar, 200μm). **(B,C)** Gross images **(B)** and H&E staining of liver tumor sections **(C)** from L2AKO mice. **(D)** Immunostaining for L2A in liver sections of a control mouse (top) and from the hepatocellular adenoma shown in **(C)**.

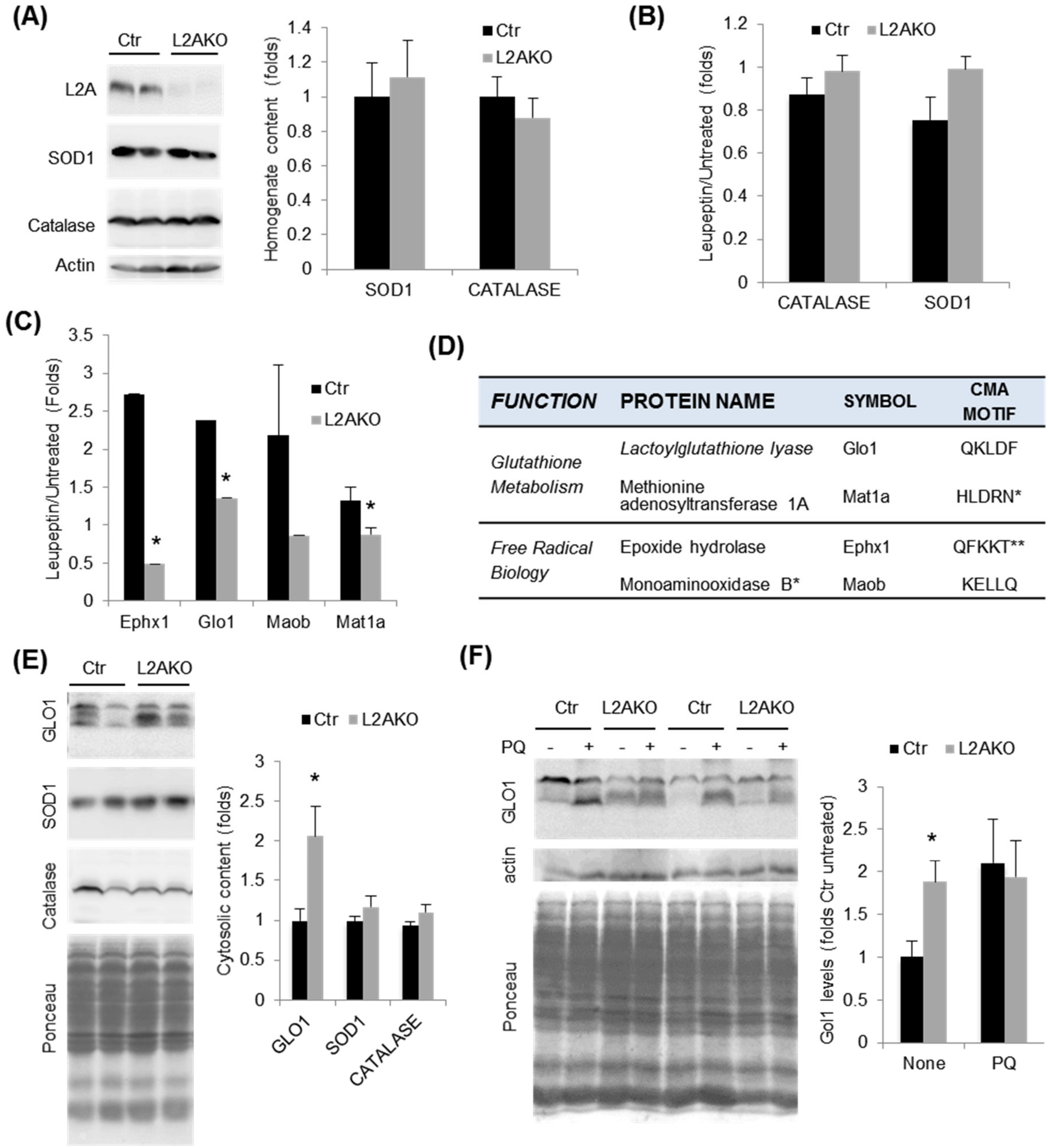


**Supplementary Figure 8. Proteins involved in the cellular oxidative stress response in livers of CMA-impaired animals. (A)** Immunoblot (IB) for the indicated proteins in livers from 4 months old control (Ctr) and Albumin-Cre:L2A$^{f/f}$ (L2AKO) mice. Right: Densitometric quantification, n=5. (**B,C)** Changes in lysosomal levels of the indicated proteins as determined by comparative proteomic analysis of lysosomes isolated from Ctr and L2AKO mice livers treated or not with leupeptin 2h prior to tissue collection, n=3. (**D**) Proteins related with the oxidative cellular response identified as CMA substrates in the proteomic analysis. CMA targeting motifs in their amino acid sequences are indicated. (E) IB of cytosolic fractions from Ctr and L2AKO mice. Right: Quantification, n =4. **(F)** IB for GLO1 in liver homogenates from Ctr and L2AKO mice treated or not with two low-dose injections of paraquat one week prior and one high-dose 24h prior to tissue collection. Right: Quantification, n=3.Ponceau staining is shown as loading control. Values are expressed as mean$\pm$s.e.m. Differences are significant for * $p<0.05$.

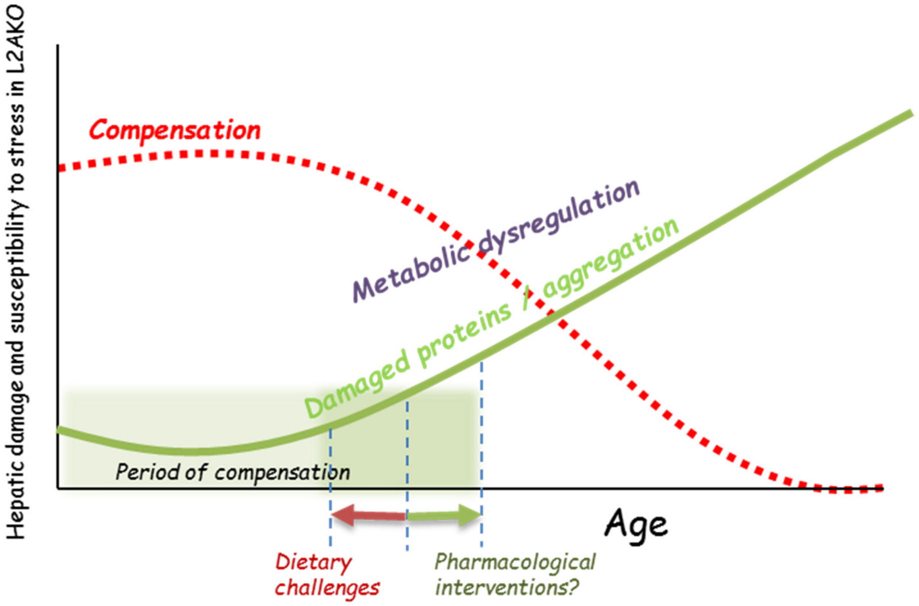


**Supplementary Figure 9. Model of the age-related changes in dual role of CMA in maintaining metabolic homeostasis and protein quality control.** In the absence of liver CMA, metabolic dysregulation is evident from an early age but problems in protein quality control are masked due to effective compensation by other proteolytic systems. However, the loss of compensatory mechanisms that occurs with age or stress (i.e. diet-induced obesity) causes a progressive worsening of the phenotype in CMA-deficient mice. The age-related hepatic functional decline is caused not only by gradual metabolic dysfunction but also by the additive deterioration in proteostasis and the ensuing accumulation of damaged proteins and aggregates. Pharmacological interventions aimed at prolonging the period of compensation may be effective in delaying the consequences of CMA failure.

## Extended Experimental Procedures

***Animal diets and treatments.*** Where indicated, mice were starved with water *ad libitum*. Mice of all genotypes were kept on a regular 12h dark/light cycle and a standard chow diet (LabDiet #5058), except for the experimental subgroup maintained on a high-fat diet (HFD, Research Diets D12492, 60% kcal% fat) for 16 weeks. Where indicated, leupeptin (20mg/kg b.w.; Fisher Scientific) or saline (in controls) was injected i.p. 2h before tissue harvesting. Acetaminophen (375mg/kg) was injected i.p. 24h before tissue collection. To induce mild oxidative stress, mice were treated with two low-dose i.p. injections of paraquat for two consecutive days one week prior to sacrifice (4mg/kg b.w.; Sigma) and one higher dose 24h before tissue harvesting (40mg/kg b.w.; Sigma). All treatments and procedures followed the National Institutes of Health guidelines for animal care.

***Hepatocyte isolation.*** Primary hepatocytes were isolated from livers of non-fasted control and liver-specific L2AKO mice by the Marion Bessin Liver Research Center Core and grown in culture as previously described (Matsuda *et al.* 2001; Edwards *et al.* 2013). Briefly, mice were anesthetized and livers were perfused and digested via the portal vein with a peristaltic pump. The livers were removed, the capsule peeled off, and the hepatocytes were dispersed in digestion media, followed by filtration through gauze. Digestion was stopped by the addition of RPMI media (Sigma-Aldrich) supplemented with 5% (v/v) Newborn Calf Serum (NCS, Hyclone), 10mM HEPES (pH 7.4), and 1% penicillin/streptomycin/fungizone (Invitrogen). Cell viability at the time of isolation was determined by trypan blue and was normally between 90-95%. The cells were plated on 35- and 60-mm dishes coated with mouse type I collagen in the above media at a density of 0.5 x $10^6$ cells or 1.5 x $10^6$ cells per dish, respectively. All cells were maintained at 37°C with 5% $CO_2$.

***Zoxazolamine-induced paralysis test.*** We analyzed the clearance time of zoxazolamine, a muscle relaxant metabolized by the liver, as an index of hepatic function. We gave mice a single i.p. injection

of zoxazolamine (150mg/kg) in olive oil, placed them on their backs and recorded the time required to regain the righting reflex after the paralysis induced by this compound (Zhang & Cuervo 2008).

***Chemicals and Antibodies.*** The source of the antibodies used in this study and dilutions used for immunoblot and immunofluorescence are as follows: LAMP-2A (Invitrogen, 1:3000), LAMP-2B (in-house, 1:3000), LAMP-2C (in-house, 1:5000), Hsc70 (Novus Biologicals, 13D3, 1:5000), LAMP-1 (hybridoma bank 1D4B or H4A3, 1:3000), Actin (Abacam, 1:10000, TFEB (Santa Cruz, 1:1000), Cathepsin D (Santa Cruz, 1:500), Atg5/12 (Novus Biologicals, 1:1000), H2AX (Cell Signaling, 1:1000), LC3 (Cell Signaling, 1:1000), Ubiquitin (Invitrogen, 1:1000), K48 Ubiquitin (Millipore, 1:1000), p62 (Biomol, 1:2000), Beclin (Novus Biologicals, 1:3000), Proteasomal subunits (Biomol, 1:500-1:1000) (hybridoma bank 1D4B or H4A3, 1:3000), CPTC-Glo-1 (hybridoma bank, 1:1000), Catalase (Abcam, 1:1000; Pierce 1:1,000) and SOD1 (Novus, 1:1000).

***Morphometric analysis and electron microscopy.*** Electron microscopy for liver was done after fixation of liver blocks (1 $mm^3$ in size) with 2% paraformaldehyde and 2% gluteraldehyde in 0.1M sodium cacodylate buffer followed by post-fixation staining with 1% osmium tetroxide and 1% uranyl acetate. After dehydratation, resin embedding and ultrathin sectioning samples were viewed on a JEOL 1200EX transmission electron microscope at 80 kV. Morphometric analysis was performed in micrographs using Image J software and classification of autophagic vacuoles was done following the standard criteria (Singh *et al.* 2009) as follows: autophagosomes were distinguished as double membrane vesicles with content of similar density as the surrounding cytosol and comprised often of recognizable cellular structures; autophagolysosomes were identified as single or partially double membrane vesicles of content of lower density than the surrounding cytosol and comprised of amorphous content or partially degraded cellular structures. In both cases, the limiting membrane had to be denuded of ribosomal particles.

***Measurement of proteasome activities.*** Catalytic activities of the proteasome were determined as previously described (Liggett *et al.* 2010; Pickering *et al.* 2010). Briefly, liver homogenates were prepared in 0.25M sucrose and diluted 1:2 in reaction buffer. Protein concentration was quantified by Lowry assay and 0.01-0.05mg of homogenate was used. The volume was brought up to 90µl in ice-cold reaction buffer followed by the addition of 10µl of substrate stock solution. Each substrate stock consisted of a 10x solution made in reaction buffer, containing AMC-tagged fluorogenic peptides to measure trypsin-like, chymotrypsin-like, or caspase-like proteasome catalytic activities. Plates were incubated at 37°C for 5 min and fluorescence readings were taken at 10 min intervals using an excitation wavelength of 350nm and an emission of 440nm for 2h. Fluorescence units were converted to moles of free AMC, with reference to an AMC standard curve of known amounts of AMC, following subtraction of background fluorescence. In some wells, Lactacystin (1µM) was added as a negative control.

***Analysis of Protein oxidation.*** Levels of oxidized proteins were determined using the OxyBlot Oxidized Protein Detection Kit (Chemicon International). Briefly, carbonyl groups of oxidized proteins were derivatized to 2,4-dinitrophenylhydrazone by reaction with 2,4-dinitrophenylhydrazine and detected by immunoblot with an antibody specific for the dinitrophenyl (DNP) derivatized groups. Fractions to be used for oxidized protein detection were supplemented with 50mM DTT after preparation from mouse livers and stored at -80°C until use. Densitometric quantification of the immunoblotted membranes was performed using unsaturated images taken in the LAS-3000 Imager and with ImageJ software (NIH). For two-dimensional oxyblots, following sample rehydration and isoelectric focusing, the strips were incubated in 2N HCl with 10mM DNP at 25°C for 20 min, washed with 2M Tris containing 30% glycerol for 15 min at RT, and incubated with DTT and Iodoacetamide equilibration buffers. After SDS-PAGE was performed to separate proteins by size, immunoblot analysis was carried out using an antibody specific for DNP moieties using the Oxyblot protein detection kit.

***Protein aggregation***. For the filter retardation assay, liver homogenates (200µg) were resuspended in 500µl of 50mM Tris pH8, 100mM NaCl, 5mM $MgCl_2$, 0.5% NP-40 and protease inhibitors. After incubation on ice for 30 min, cells were centrifuged at 16,000g for 10 min at 4°C to pellet aggregated proteins as previously described(Massey *et al.* 2008). Pellets were resuspended in 200µl of Tris Buffer with 4% SDS and 100mM DTT, vortexed, and boiled at 100°C for 5 min. The samples were filtered through a 0.45µm nitrocellulose membrane in a BioDot Blot apparatus (Bio-Rad). After filtration, the aggregates caught in the membrane were assessed by immunoblot using antibodies against ubiquitinated proteins.

***In vivo measurement of reactive oxygen species***. Mice were anesthetized with an i.p. injection of a mixture of ketamine and xylazine (6.6:1) and imaged using an *In Vivo* Imaging System (IVIS, Kodak Image Station 400MM PRO, Carestream Health) before any treatments to assess baseline autofluorescence. Mice were treated by tail vein injection with a fluorogenic probe that detects reactive oxygen species (ROS), purchased from Molecular Probes (CellROX Deep Red Reagent C) (Scharf *et al.* 2013). Mice were imaged 20 min after injection, followed by opening of their abdominal cavity to image their organs *in situ*. Lastly, organs were dissected and removed from the body and imaged *ex vivo*. For fluorescence imaging, the machine was configured for 640 nm excitation and 664 nm emission, 3 min exposure, 2x2 binning and f-stop 2.5. The acquired images were analyzed with the Carestream MI Application 5.0.2.30 software (Carestream Health)

***Cell viability.*** Cell viability was determined by using the CellTiter-Blue Cell Viability Assay kit from Promega (Madison, WI). Briefly, cells were seeded at 30,000 cells per well in a 96-well plate and various treatments were performed. At the indicated times post-stress and post-recovery from stress, cells were treated with 20µl of the CellTiter Blue Reagent and incubated at 37°C. Measurements were done at 30 min or 1h intervals for up to 6h in which fluorescence readings were taken using an

excitation wavelength of 540nm and emission wavelength at 590nm. Results were plotted as percent viability of control (no treatments).

***Isolation of nuclear fractions.*** Nuclear fractions were isolated with the NE-PER lysis Kit (Pierce) following manufactures' directions or by a NP-40 based lysis protocol (Cuervo *et al.* 1998). Briefly, cells were washed with PBS and lysed by incubation on ice in a NP-40 buffer (50mM Tris-HCl, pH 7.6; 150mM NaCl; 20mM NaF, 1mM EDTA, 1mM EGTA, 0.5% NP-40, 10% Glycerol). The nuclear pellet was collected by centrifugation and lysed in a high-salt buffer (50mM Tris-HCl, pH 7.6; 500mM NaCl; 20mM NaF, 1mM EDTA, 1mM EGTA, 1% NP-40, 10% Glycerol) by sonication with 3 burst/5 sec each. The nuclear pellet was collected by centrifugation a 15,000g for 5 min. Enrichment and purity of the fractions were evaluated by analysis of levels of γH2A (nuclear) and GAPDH (cytosol) in the samples.

**Quantitative Proteomics.** Comparative proteomics of lysosomes from Ctr and L2AKO mice was performed as described before (Schneider *et al.* 2014). Briefly, lysosomes active for CMA were isolated from 24 hour-starved Ctr and L2AKO mice treated or not with leupeptin two hours before isolation. Three different sets of lysosomes from three different animals were separately analyzed for purity, integrity, electrophoretic patterning and enrichment in markers of CMA lysosomes by immunoblot. Quantitative proteomics analysis was performed using iTRAQ multiplex (Applied Biomics) in the three animals under the four different conditions: Ctr mice untreated, Ctr mice treated with leupeptin, L2AKO mice untreated and L2AKO mice treated with leupeptin. For each protein hit the average ratio(s) for the protein, the number of peptide ratios that contributed and the geometric standard deviation were determined. Values in the three experimental groups were compared to untreated Ctr and are represented as the average of folds (lysosomes isolated from untreated Ctr mice are given a value of 1). CMA substrate proteins were defined as those for which leupeptin

treatment resulted in increase in lysosomal levels >20% and with a reduction in leupeptin response of >20% in the L2AKO.

***Other methods.*** Cell viability was determined by using the CellTiter-Blue Cell Viability Assay kit from Promega (Madison, WI). Protein concentration was determined by the Lowry method(Lowry *et al.* 1951) using bovine serum albumin as a standard. For immunoblotting, protein concentration was determined by the Lowry method(Lowry *et al.* 1951) using bovine serum albumin as a standard. After SDS-PAGE, gels were transferred to nitrocellulose membranes using a Mini-TransBlot SD wet transfer cell (Bio-Rad, Richmond, VA) and immunoblotting was performed following standard procedures(Towbin *et al.* 1979). Proteins recognized by the specific antibodies were visualized by chemiluminescence (RenaissanceR; PerkinElmer Life and Analytical Sciences) using peroxidase conjugated secondary antibodies in a LAS-3000 Imaging System (Fujifilm). Densitometric quantification of the immunoblotted membranes was performed with ImageJ software. Densitometric quantification of the immunoblotted proteins was performed from the TIFF images generated by detection of the chemiluminescent signal after subtraction of background. Although different exposures of each membrane were captured, we quantified those in which none of the bands were saturated. If in order to visualize low abundant proteins, we needed to use exposure times in which some bands were saturated. In this case, two different exposure times were quantified and a common unsaturated band in both exposures was utilized for normalization. Where applicable, purified protein was loaded on the same gel as an input and used for normalization across experiments.

***Statistical analysis.*** All numerical results are reported as mean + standard error of the mean (s.e.m.) and represent data from a minimum of three independent experiments unless otherwise stated. We determined the statistical significance of the difference between experimental groups in instances of single comparisons by the two-tailed unpaired Student's t-test with the Sigma Plot

software (Jandel Scientific). In instances of multiple means comparisons, we used one-way analysis of variance (ANOVA) followed by the Bonferroni post-hoc test to determine statistical significance ($p<0.05$). Statistical analysis was performed in all the assays and significant differences are noted by graphical representations.

**Additional References**

#

Cuervo AM, Hu W, Lim B, Dice JF (1998). IkappaB is a substrate for a selective pathway of lysosomal proteolysis. *Mol Biol Cell*. **9**, 1995-2010.

Edwards M, Houseman L, Phillips IR, Shephard EA (2013). Isolation of mouse hepatocytes. *Methods in molecular biology*. **987**, 283-293.

Liggett A, Crawford LJ, Walker B, Morris TC, Irvine AE (2010). Methods for measuring proteasome activity: current limitations and future developments. *Leuk Res*. **34**, 1403-1409.

Lowry OH, Rosebrough NJ, Farr AL, Randall RJ (1951). Protein measurement with the Folin phenol reagent. *The Journal of biological chemistry*. **193**, 265-275.

Massey AC, Follenzi A, Kiffin R, Zhang C, Cuervo AM (2008). Early cellular changes after blockage of chaperone-mediated autophagy. *Autophagy*. **4**, 442-456.

Matsuda M, Korn BS, Hammer RE, Moon YA, Komuro R, Horton JD, Goldstein JL, Brown MS, Shimomura I (2001). SREBP cleavage-activating protein (SCAP) is required for increased lipid synthesis in liver induced by cholesterol deprivation and insulin elevation. *Genes Dev*. **15**, 1206-1216.

Pickering AM, Koop AL, Teoh CY, Ermak G, Grune T, Davies KJ (2010). The immunoproteasome, the 20S proteasome and the PA28alphabeta proteasome regulator are oxidative-stress-adaptive proteolytic complexes. *Biochem J*. **432**, 585-594.

Scharf B, Clement CC, Yodmuang S, Urbanska AM, Suadicani SO, Aphkhazava D, Thi MM, Perino G, Hardin JA, Cobelli N, Vunjak-Novakovic G, Santambrogio L (2013). Age-related

carbonylation of fibrocartilage structural proteins drives tissue degenerative modification. *Chemistry & biology*. **20**, 922-934.

Schneider JL, Suh Y, Cuervo AM (2014). Deficient Chaperone-Mediated Autophagy in Liver Leads to Metabolic Dysregulation. *Cell metabolism*. **DOI: 10.1016/j.cmet.2014.06.009**.

Singh R, Kaushik S, Wang Y, Xiang Y, Novak I, Komatsu M, Tanaka K, Cuervo AM, Czaja MJ (2009). Autophagy regulates lipid metabolism. *Nature*. **458**, 1131-1135.

Towbin H, Staehelin T, Gordon J (1979). Electrophoretic transfer of proteins from polyacrylamide to nitrocellulose sheets: procedure and some applications. *Proc Natl Acad Sci*. **76**, 4350-4354.

Zhang C, Cuervo AM (2008). Restoration of chaperone-mediated autophagy in aging liver improves cellular maintenance and hepatic function. *Nat Med*. **14**, 959-965.
